# SitkaNet: A low-cost, distributed sensor network for landslide monitoring and study

**DOI:** 10.1016/j.ohx.2021.e00191

**Published:** 2021-03-11

**Authors:** Max Chu, Annette Patton, Josh Roering, Cora Siebert, John Selker, Cara Walter, Chet Udell

**Affiliations:** a OPEnS Lab, Oregon State University, Corvallis, OR, United States; b Department of Earth Sciences, University of Oregon, Eugene, OR, United States; c Sitka Sound Science Center, Sitka, AK, United States; d Department of Biological & Ecological Engineering, Oregon State University, Corvallis, OR, United States

**Keywords:** Data logging, Landslide, Monitoring, Near real time data, Arduino, Soil moisture, Rainfall, Humidity, Barometric pressure, Piezometer, Wireless data transmission, LoRa

## Abstract

Landslides threaten the infrastructure and safety of communities. Soil conditions can predict landslide threat, but the cost and complexity of sensing systems for documenting hazardous conditions across a heterogeneous spatial area prevent widespread utilization. The SitkaNet system is a low-cost, easier to install alternative that allows for numerous sites to be monitored with real-time reporting and expands the accessibility of data-driven landslide forecasting. Using a combination of industry-proven sensors and cheaper alternatives, each SitkaNet node can measure the rainfall, six soil moisture sensors at varying depths, water table, atmospheric pressure, humidity, and temperature at each site for less than one-fifth the cost of existing solutions (<$1000/node). The SitkaNet nodes transmit data wirelessly at five-minute intervals over LoRa network to an Ethernet connected hub instead of more traditional on-site cellular or satellite methods. The node electronics are packaged with 3D printed components in a small waterproof case mounted on a hand-driven well-point utilized for the water level measurement. Each node is intended for operation for more than six months on a lithium-ion battery pack: no solar panel is needed, so amenable to low-light sites. The installation process is streamlined which allows for a node to be installed in less than a day compared to multi-day procedures required by other systems.

## Graphical abstract

**Figure:**
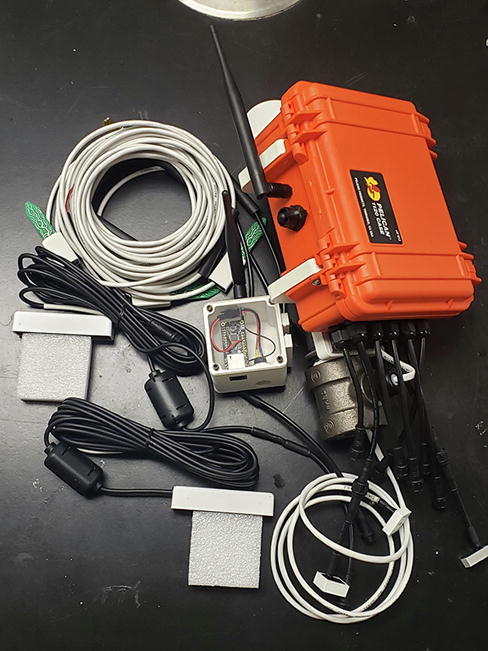

**Specifications table**

**Table:** 

Hardware name	SitkaNet
Subject area	Environmental, Planetary and Agricultural Sciences
Hardware type	Field measurements and sensors
Open Source License	CERN Open Hardware License GNU General Public License v3.0
Cost of Hardware	$940 per node, $165 per hub
Source File Repository	*https://osf.io/497gt/* *https://doi.org/10.17605/OSF.IO/497GT*

## Hardware in context

Communities throughout the United States struggle to manage significant landslide risk. Landslides are low frequency, high consequence, hard-to-predict events, the response to which can often prove economically costly and socially disruptive. While the science of landslide prediction has improved in recent years, there remains a significant disconnect between this science and effective responses, such as warning systems and land use policies [1]. Effective landslide risk management requires an integrated understanding of the geoscience of the natural hazard and accessible, cost-effective options for warning and response. Rainfall-induced slope failures are most commonly responsible for casualties and damage to infrastructure, and early warning systems have been employed to minimize exposure [2], [3]. To forecast conditions conducive to slope failure, these systems often account for the offsetting effects of rainfall intensity and antecedent soil moisture [4]. Put simply, low-moisture soils require high intensity storms to trigger sliding, whereas wetter soils can fail during lower intensity storms. Because the accurate, affordable, and timely measurement of soil moisture has traditionally been infeasible, the vast majority of landslide warning systems use average rainfall data (e.g., two weeks to two months) as a proxy [5]. However, rainfall-based soil moisture proxies based on sparse data are a poor substitute for in-situ soil moisture data, which limits the efficacy of many warning systems [6].

The recent emergence of low-cost, low-power sensors for environmental monitoring (e.g. [7]) offers tremendous potential for improving landslide warning systems [8]. Rather than rely on proxies or a small number of soil moisture measurements, dense wireless sensor networks and long-range telemetry provide the means to accurately monitor soil moisture across vast areas and improve landslide warning systems (e.g. [9], [10], [11]). A recent study demonstrated the utility of soil moisture sensors for a slide-prone railway corridor in the Seattle Puget Sound region [12]. Unfortunately, the existing technology to monitor hillslope hydrology is prohibitively expensive ($8000–$10,000 per site, not including maintenance and analysis costs) and many communities lack the economic and technical support to establish long-term monitoring efforts. Similar to recent advances in seismology that capitalize on large seismometer arrays [13], soil and hydrologic measurements with inexpensive, portable sensors will facilitate vast improvements in our ability to characterize landslide potential in heterogeneous landscapes. The monitoring system described in this study is a low-cost, easy-to-use alternative for data-driven hillslope monitoring. Implementation of this system is feasible for individual communities, which expands access to data-driven hydrologic monitoring. In particular, small communities will be able to implement their own landslide warning systems rather than rely on strained state and federal resources [1] and can tailor risk prevention efforts to meet their individual needs.

**Fig. 1:**
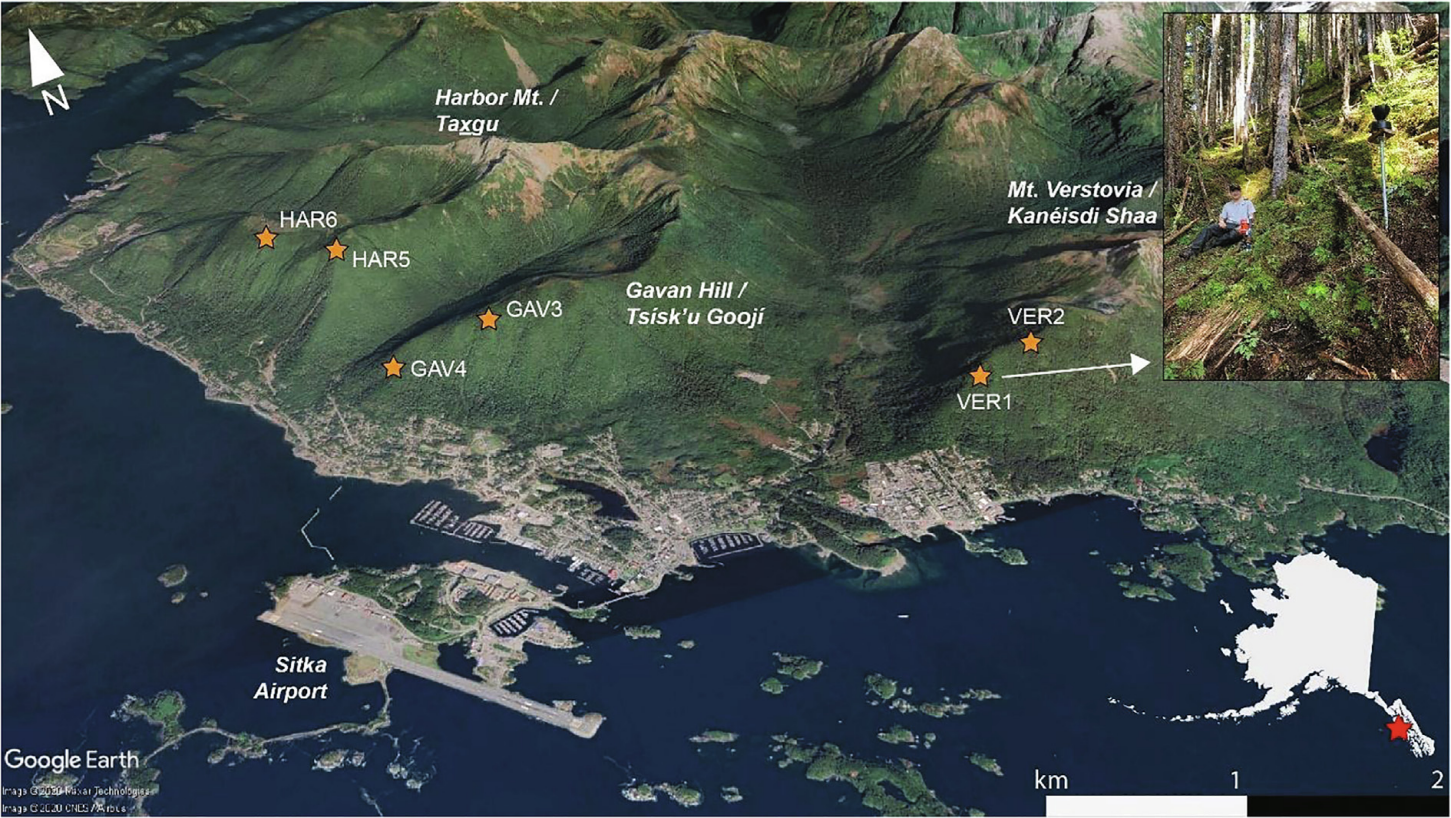
Map of preliminary sensor network in Sitka, Alaska. Sensor sites are shown as orange stars. Monitoring locations were selected based on landslide potential and geomorphic characteristics. This network of nodes documents conditions at sites that may experience landslides and pose hazards to populated areas of Sitka. (For interpretation of the references to color in this figure legend, the reader is referred to the web version of this article.)

A sensor network was deployed based on the specifications outlined in this study in Sitka, Alaska, in August 2020 (Fig. 1). Sitka presents a unique opportunity for implementing and testing a new approach to landslide risk management. The town occupies a narrow strip of flat coastal land on Alaska’s Baranof Island pressed against the dramatic, steep slopes of the Tongass National Forest. Ancient and recent landslide chutes litter these mountains, whose soils are made even more complex by an uneven distribution of volcanic ash from nearby Mt. Edgecumbe’s eruption around 2200 BCE [14]. The relatively frequent landslides on Baranof Island are documented in a historical dataset curated by the US Forest Service (USFS), but Sitkans are newly focused on their landslide risk. In August 2015, three landslides struck the town, one killing three people; forty-five landslides were documented in the surrounding area that day [15]. Recent hazard assessments and runout models demonstrate that large areas of the town, including the elementary school, are at risk from landslides [16], [17], [18].

Each of the six nodes was deployed in a topographic depression (colluvial hollow) where shallow landslides and debris flows may initiate during high-intensity precipitation [19]. Soil moisture sensor depth varied from site to site depending on total soil depth, which ranged from 0.5 m to >1 m, with the well piezometer deployed at the maximum soil depth or the well depth, 0.95 m. Soil moisture sensors were located at variable depths according to local soil horizons and geomorphic stratigraphy in order to capture hydrologic responses along geomorphic boundaries. These depths are useful characterizations for landslide initiation because shallow-seated landslides typically occur along geomorphic failure planes (such as the soil–bedrock interface) or at critical soil depths on the order of 1 m [20]. For example, at four of the six sites, sensor depths were chosen to characterize the A horizon in volcanic soil, B horizon in volcanic soil, and coarse colluvium. At two of the six sites, sensor depths were chosen to characterize the A horizon in glacial soil, the B horizon in glacial soil, and relatively un-weathered glacial sediment.

## Hardware description

**Fig. 2:**
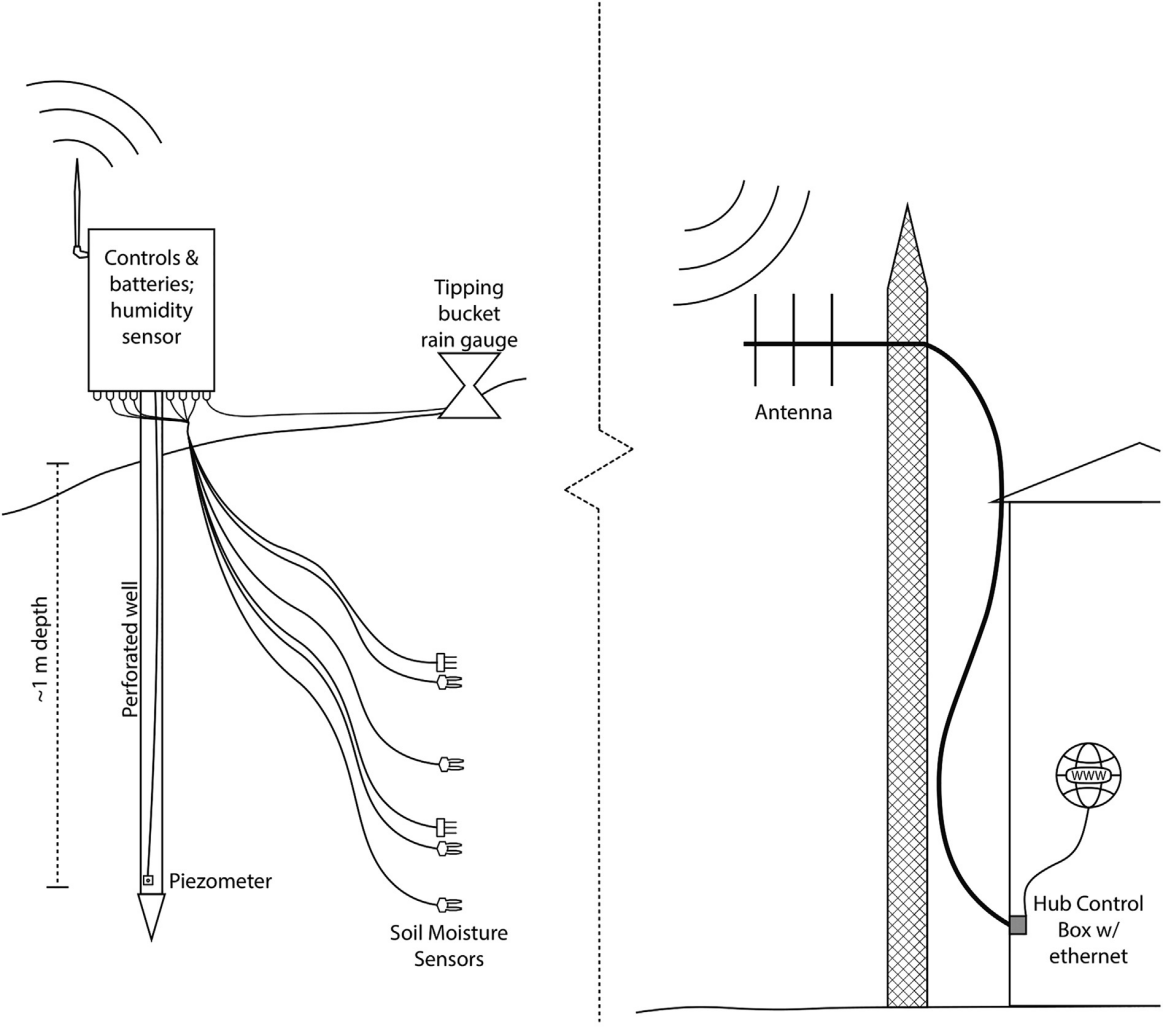
Sketch of main components of SitkaNet node (left) and hub (right), showing the configuration of each node, the types of sensors at each site, and wireless connectivity for remote real-time data access.

The currently deployed SitkaNet system is composed of six sensor nodes transmitting data wirelessly to two centrally located internet-connected hubs (Fig. 2), which then upload the data in near real time to an online spreadsheet. While there are no hardware limitations to prevent mesh type networking of this system, our firmware only supports star-type network structures, due to the power consumption needs of nodes in continuous listening mode and complexities of a heartbeat refresh pulse. The nodes and hubs utilize the Arduino compatible Adafruit Feather M0 microprocessor and communicate using the LoRa radio network: a low power and free alternative to power hungry and expensive cellular or satellite communications. The microprocessor is programmed to collect and relay data from the sensors to the hubs at specified intervals.

The C/C++ code for the microprocessors is based on the existing, open-source LOOM project. LOOM provides a foundation to easily build sensor systems without having to code much of the low-level operations from scratch. It also allows the system to be easily adapted to different sensing applications. LOOM supports a number of I^2^C and SDI-12 sensors, which can be hot-swapped into the system, wherein the system could be used to measure any number of variables, including temperature, relative humidity, solar radiation, ultrasonic distance (for snow depth measurements), etc. A full list of supported sensors can be found here.

### Nodes

Each node consists of 11 sensors which monitor soil moisture at different soil depths, rainfall, and atmospheric conditions at five-minute intervals (Table 1). The Teros 11 sensors are industry standard sensors and were selected to provide a baseline measurement to calibrate the cheaper STEMMA soil moisture sensors at three heights. The accelerometer is used to detect any acceleration over 3G, which would indicate a landslide had occurred in the area containing the node. The SHT31D humidity sensor and one of the MS580302 pressure sensors measure the atmospheric conditions around the node. The second pressure sensor is used as a piezometer at the bottom of a well.

**Table 1:** Sensors included in each node.

Brand	Model	Quantity	Purpose
METER Group	Teros 11	3	Industry standard soil moisture sensor
Adafruit	STEMMA Soil Sensor	3	Low cost soil moisture sensor
TE Connectivity	MS580302ba01-00	2	Pressure and temperature
Adafruit	SHT31D	1	Humidity
Adafruit	MMA8451	1	Accelerometer
Davis Instruments	Aerocone	1	Rainfall

The Teros 11 sensors communicate with the microprocessor over the SDI-12 protocol and the rest of the sensors are connected using I^2^C through an I^2^C multiplexer mounted on a custom PCB, I2C MUX, to facilitate connection to the microprocessor.

The Hypnos is a custom printed circuit board that connects a DS3231 real time clock (RTC), microSD card, and switching power control to the sensor system [21]. The RTC provides timestamps for the data, and data is backed up the microSD card in case of issues with the wireless data transmission. The third main function of the Hypnos board is to allow the microprocessor to turn off the power to the sensors.

In between the five-minute intervals, the microprocessor enters a “sleep mode” wherein the Hypnos board shuts down power to the sensors to prolong battery life. Each node is powered by five 3.3 V 6600mAh lithium ion batteries connected in parallel via a custom PCB, JST parallel. This provides the node with an expected battery life of over six months. Appendix A shows a power consumption budget for a node.

One of the goals of the SitkaNet project was to ruggedize cheap, readily available hardware to reduce costs and dependence on expensive and proprietary products. Except for the Teros 11 sensors, the selected electronics were not necessarily designed or advertised for use in harsh environments. Testing and characterization of “hobbyist-grade” hardware offers similar functionality to industry proven devices at much lower costs. The control electronics and the accelerometer are protected inside a waterproof case, and any sensors exposed to the elements were modified to be physically robust and waterproof using 3D printed cases and potting compound.

The node electronics, batteries, accelerometer, humidity sensor, and one pressure sensor are housed inside a modified IP67 rated Pelican 1120 case and attached to the top of a well pipe. This case protects the electronics from physical damage such as falling rocks, rain or snow, UV exposure, and wildlife. The microprocessor, Hypnos board, microSD card extension, and I^2^C multiplexer are mounted to a custom fit 3D printed insert inside the case. Modifications to the case include holes drilled to pass through sensor cables, the antenna connector, and a micro-USB connector, which have all been sealed with O-rings to maintain the waterproofness of the enclosure. The sensor cables are routed through cable grips attached to the bottom of the case, which provide strain relief and keeps the case watertight. The Pelican case uses 3D-printed brackets and U-bolts to mount to the side of a well pipe, which is driven into the soil and is also the well for piezometer.

The hub consists of a Feather M0 microprocessor with an integrated LoRa module and an Ethernet adapter. Data from the nodes is received over LoRa and the hub process and uploads the data to the spreadsheet. The electronics are mounted in a 3D printed case and is powered from a 5 V power supply.

Existing sensing and datalogging systems are geared toward a broad assortment of diverse applications. This results in hardware and software that is overly expensive and bulky for most functions. The SitkaNet system is focused on landslides and results in a much more cost effective and compact solution, which in turn allows for more sites to be instrumented. A SitkaNet node is compact and lightweight enough to be installed by as few as two people in under a few hours. Existing solutions take twice the number of people and twice the amount of time.

• Low-cost, compact sensor network for in-situ sensing
• Robust, low-maintenance, and easy to install
• Near real time data transmission

## Design files

### Design files summary

Node Design Files

**Table:** 

Design file name	File type	Open source license	Location of the file
Case Insert	STL	CERN Open Hardware License	https://osf.io/3pvmj/
Pelican Case Mount Top	STL	CERN Open Hardware License	https://osf.io/ysgcv/
Pelican Case Mount Bottom	STL	CERN Open Hardware License	https://osf.io/754tq/
MS5803 Case	STL	CERN Open Hardware License	https://osf.io/czaxt/
SHT31D Case	STL	CERN Open Hardware License	https://osf.io/jv6e9/
STEMMA Case	STL	CERN Open Hardware License	https://osf.io/tjqzd/
I2C-MUX	BRD	CERN Open Hardware License	https://osf.io/djebn/
MS5803-02BA	BRD	CERN Open Hardware License	https://osf.io/e8vqd/
Jst parallel	BRD	CERN Open Hardware License	https://osf.io/djebn/
Node	ino	GNU General Public License v3.0	https://osf.io/43cpb/

Hub Design Files

**Table:** 

Design file name	File type	Open source license	Location of the file
Hub Case	STL	CERN Open Hardware License	https://osf.io/s6uhz/
Hub Lid	DXF	CERN Open Hardware License	https://osf.io/zgpsk/
Hub	ino	GNU General Public License v3.0	https://osf.io/pa3d2/

**Case Insert:** STL file for 3D printing the insert which holds the electronics inside the Pelican case.

**Pelican Case Mount Top:** STL file for 3D printing the top part of the Pelican case mount.

**Pelican Case Mount Bottom:** STL file for 3D printing the bottom part of the Pelican case mount.

**MS5803 Case:** STL file for 3D printing the MS5803 pressure sensor protective case.

**SHT31D Case:** STL file for 3D printing the SHT31D humidity sensor protective case.

**STEMMA Case:** STL file for 3D printing the STEMMA soil water content sensor protective case.

**I2C-MUX:** BRD file for creating the I^2^C multiplexer PCB

**MS5803-02BA:** BRD file for creating the MS5803 pressure sensor PCB

**Jst parallel:** BRD file for creating the parallel battery connection PCB

**Node:** Arduino file used as firmware for a node microcontroller

**Hub Case:** STL file for 3D printing the hub enclosure.

**Hub Lid:** DXF file for laser cutting the lid of the hub enclosure.

**Hub:** Arduino file used as firmware for a hub microcontroller

## Bill of materials

### Node BOM

**Table:** 

Designator	Component	Number	Cost per unit -currency	Total cost - currency	Source of materials	Material type
PVC Cap	1 1/4″ PVC cap	1	$1.20	$1.20	McMaster	Polymer
PVC Pipe	1–1/4″ 1ft PVC pipe	1	$10.37	$10.37	McMaster-Carr	Polymer
U-bolt	U-Bolt	2	$4.85	$9.70	McMaster	Metal
Pipe Tee	1–1/4″ pipe tee	1	$15.43	$15.43	McMaster	Metal
3/4 NPT Cable Grip	3/4 NPT cable grip	1	$4.61	$4.61	McMaster	Polymer
M2 Insert	M2 Taper Heat-Set Inserts	8^(A)^	$0.15	$1.20	McMaster-Carr	Metal
M2 Screw	M2 × 0.4 mm stainless steel socket head screw	8^(B)^	$0.17	$1.36	McMaster-Carr	Metal
1/4–20 Screw	1/4″-20 x5/8″ Stainless Steel Button Head Screw	2^(B)^	$0.13	$0.26	McMaster-Carr	Metal
PG7 Cord Grip	PG7 cord grip	10^(C)^	$0.39	$3.99	Amazon	Polymer
Pelican Case	Pelican 1120 case (orange)	1	$39.00	$39.00	Amazon	Polymer
Potting Compound	Potting Compound 50 mL	1	$6.99	$6.99	Amazon	Polymer
MicroSD Card	16 GB MicroSD Card	1	$13.95	$13.95	Amazon	Semiconductor
MicroSD Card Extension	MicroSd card extension	1	$5.79	$5.79	Amazon	Non-specific
JST XH Connector	JST XH connectors	1	$9.87	$9.87	Amazon	Non-specific
JST SM Connector	JST SM connector	1	$12.99	$12.99	Amazon	Non-specific
Ribbon Cable	Ribbon Cable	1	$6.99	$6.99	Amazon	Non-specific
USB Extension	Waterproof panel mount micro-USB connector	1	$14.35	$14.35	USBFirewire	Non-specific
USB Extension Cap	Sealing cap for panel mount micro-USB connector	1	$2.50	$2.50	USBFirewire	Non-specific
Ethernet Cable	75ft ethernet cable	2	$9.69	$19.38	Monoprice	Non-specific
1/16″ Heat shrink	1/16″ heat shrink tubing	1	$0.38	$0.38	Digikey	Polymer
5/16″ Heat shrink	5/16″ adhesive lined heat shrink	1	$2.61	$2.61	Digikey	Polymer
10 k Resistor	10 k 0805 resistor	4	$0.11	$0.44	Digikey	Non-specific
100nF Capacitor	100nF 0805 capacitor	2	$0.10	$0.20	Digikey	Non-specific
u.Fl Connector	u.Fl connector	1	$0.75	$0.75	Mouser	Non-specific
Waterproof Cable	Waterproof 4 pin cable set	10	$2.50	$25.00	Mouser	Non-specific
MMA8451 Accelerometer	MMA8451 Accelerometer	1	$7.95	$7.95	Mouser	Non-specific
Ms5803	Ms580302ba pressure sensor	2	$10.72	$21.44	Mouser	Non-specific
Sht31D	Sht31D humidity sensor	1	$13.95	$13.95	Mouser	Non-specific
STEMMA	Adafruit STEMMA Capacitive soil moisture sensor	4	$7.50	$30.00	Mouser	Non-specific
Feather M0	Feather M0 LoRa	1	$34.95	$34.95	Mouser	Non-specific
Battery	6600mAh Lithium Battery	5	$29.50	$147.50	Mouser	Non-specific
Feather Doubler	Feather doubler	1	$7.50	$7.50	Mouser	Non-specific
Female Headers	Female headers	3	$1.50	$4.50	Mouser	Non-specific
TCA9548A	TCA9548A multiplexer	1	$6.95	$6.95	Mouser	Non-specific
Antenna	LoRa Antenna	1	$15.93	$15.93	Mouser	Non-specific
Coin Cell	CR1220 coin cell battery	1	$0.95	$0.95	Mouser	Non-specific
Teros	Teros 11	3	$190.00	$570.00	Meter Group	Non-specific
Tipping Bucket	Tipping Bucket	1	$95.00	$95.00	Davis Instruments	Non-specific
MS5803 PCB	Smart rock MS5803 PCB	2	$0.48	$0.96	https://osf.io/e8vqd/	Non-specific
I2C multiplexer PCB	I2C multiplexer PCB	1	$5.77	$5.77	https://osf.io/djebn/	Non-specific
Battery PCB	JST parallel PCB	1	$1.98	$1.98	https://osf.io/djebn/	Non-specific
Hypnos	Hypnos Board	1	$33.00	$33.00	OPEnS Lab	Non-specific

### Hub BOM

**Table:** 

Designator	Component	Number	Cost per unit -currency	Total cost -currency	Source of materials	Material type
Feather M0	Feather M0 LoRa	1	$34.95	$34.95	Mouser	Non-specific
Ethernet Featherwing	Ethernet Featherwing	1	$19.95	$19.95	Mouser	Non-specific
DC Barrel Jack	Panel Mount 2.1 mm DC barrel jack	1	$2.95	$2.95	Mouser	Non-specific
Power Supply	5 V 2A switching power supply	1	$7.95	$7.95	Mouser	Non-specific
Yagi Antenna	900 MHz 9 dB Yagi antenna	1			Campbell Scientific	Non-specific
RF Cable	LOW-400-DB RF cable (70 ft.)	1	$83.80	$83.80	TXM	Non-specific
Antenna Adapter Cable	RP-SMA to u.Fl adapter cable	1	$15.93	$15.93	Mouser	Non-specific
M3 inserts	M3 Tapered Heat-Set Inserts	4^(A)^	$0.13	$0.52	McMaster-Carr	Metal
M3 Screws	M3 × 0.5, 8 mm Stainless Steel Socket Head Screw	4^(A)^	$0.05	$0.20	McMaster-Carr	Metal

Pricing Notes:

(A) Sold in packs of 100
(B) Sold in packs of 50
(C) Sold in packs of 20

### 3D printed/laser cut components

• Approximate cost of 3D printed components printed on Fusion3 F400 using 3DXTech ASA filament.
• Approximate cost of laser cut components cut from 1/8″ clear acrylic using BODOR Laser Cutter.

#### Node components

**Table:** 

Designator	Component	Number	Cost per unit -currency	Total cost - currency	Source of materials	Material type
Case Insert	Case Insert	1	$3.18	$3.18	3DXTECH	Polymer
Pelican Case Mount Top	Pelican Case Mount Top	1	$2.91	$2.91	3DXTECH	Polymer
Pelican Case Mount Bottom	Pelican Case Mount Bottom	1	$2.91	$2.91	3DXTECH	Polymer
MS5803 Case	MS5803 Case	2	$0.13	$0.26	3DXTECH	Polymer
SHT31D Case	SHT31D Case	1	$0.14	$0.14	3DXTECH	Polymer
STEMMA Case	STEMMA Case	4	$0.12	$0.48	3DXTECH	Polymer

#### Hub components

**Table:** 

Designator	Component	Number	Cost per unit -currency	Total cost - currency	Source of materials	Material type
Hub Case	Hub Case	1	$2.37	$2.37	3DXTECH	Polymer
Hub Lid	Hub Lid	1	$1.00	$1.00	3DXTECH	Polymer

## Build instructions

### Node

#### Electrical assembly

##### Microprocessor

**Fig. 3:**
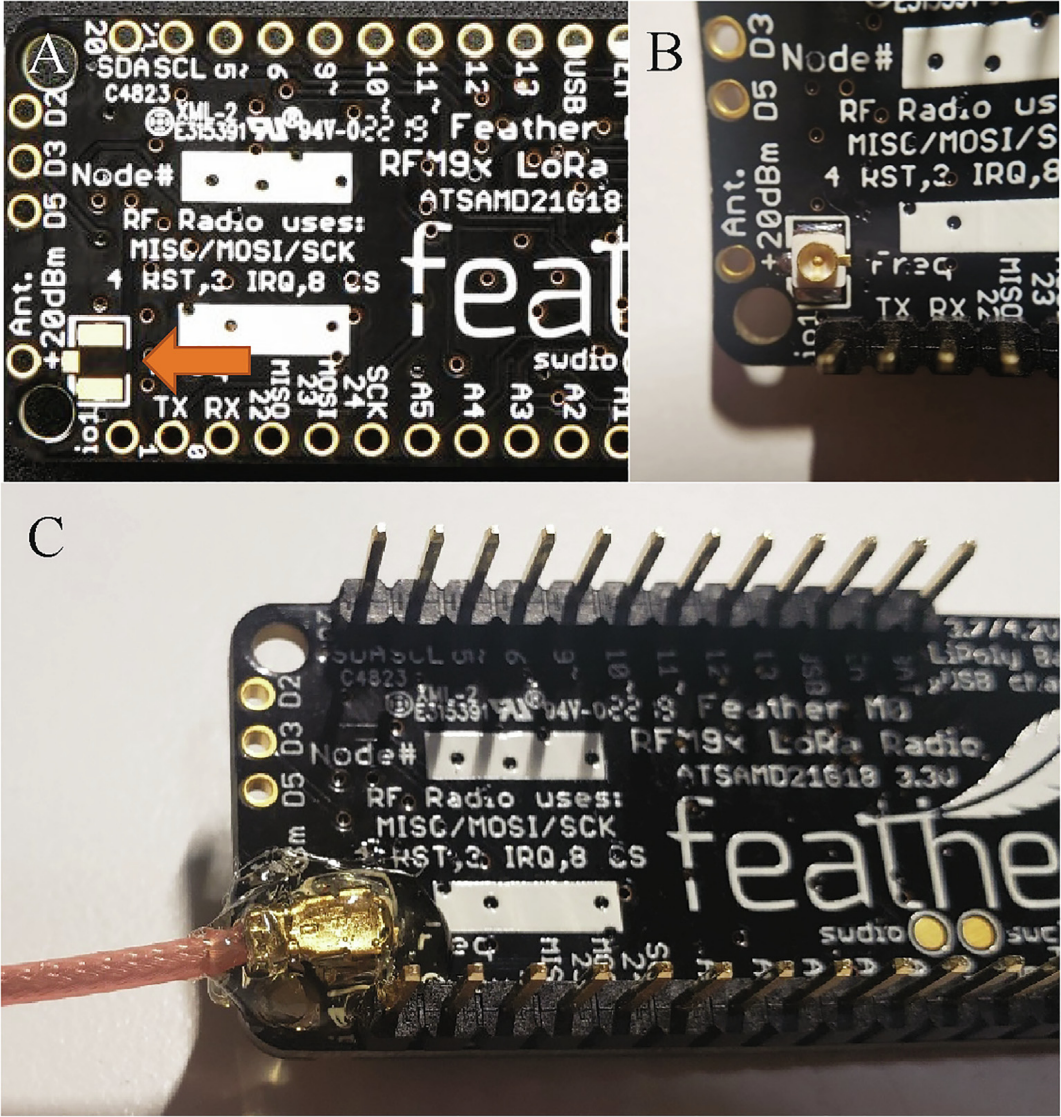
(A) Unsoldered Feather M0 with orange arrow pointing to solder pad for the u.Fl connector. (B) Feather M0 with u.Fl connector and headers soldered. (C) Male header pins soldered, antenna adapter cable attached, and hot glue applied to the connection (B). (For interpretation of the references to color in this figure legend, the reader is referred to the web version of this article.)

The microprocessor requires one sets of headers and an antenna connector. On the underside of the Feather M0, solder the male headers included with the Feather M0 to the board (Fig. 3C) and the u.Fl connector to the spot marked Ant. +20dBm. (Fig. 3A, 3B). Attach the antenna adapter cable to the u.Fl connector and add hot glue to the u.Fl connector (Fig. 3C).

##### Hypnos

**Fig. 4:**
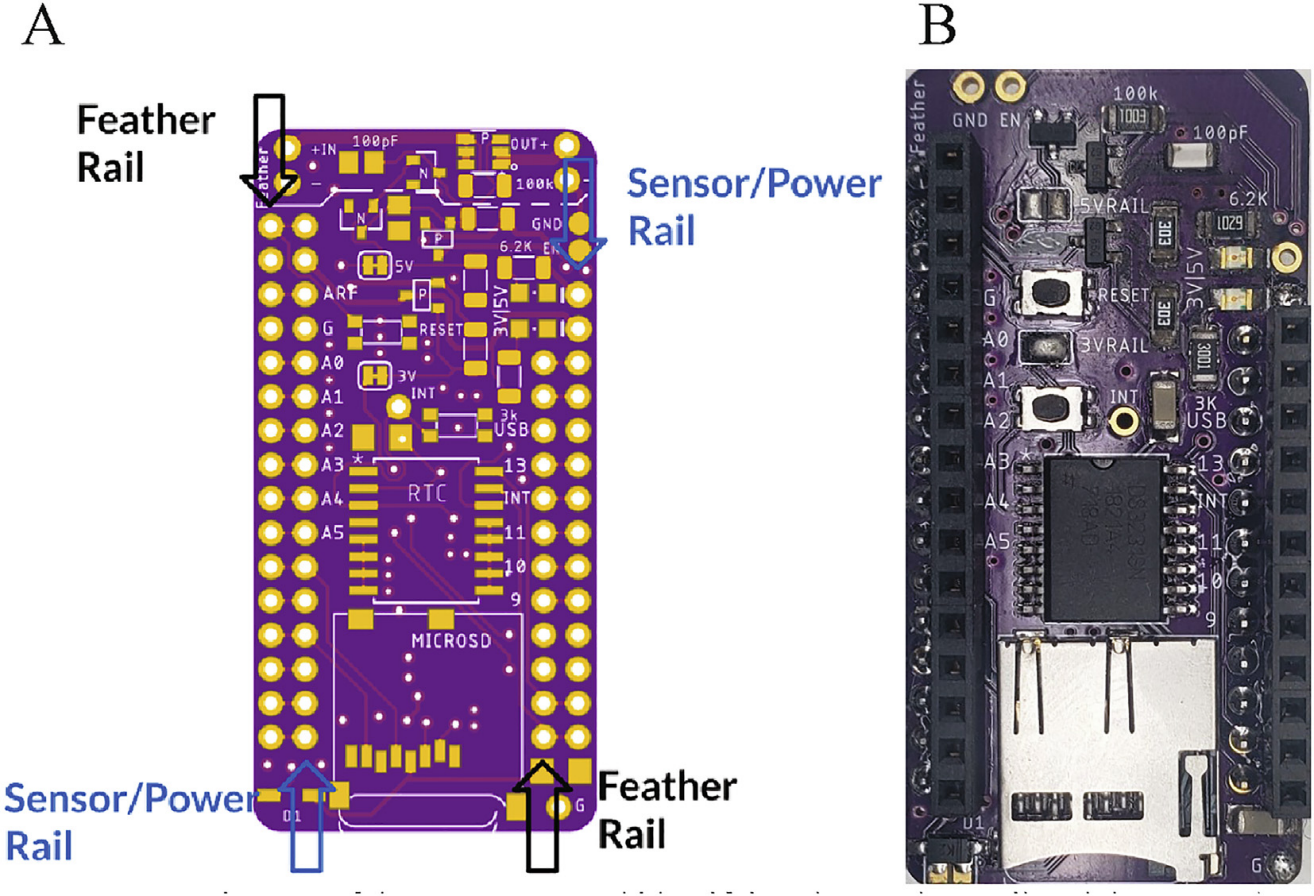
(A) Diagram of the Hypnos Board identifying the Feather Rail and the Sensor/Power Rail. (B) Hypnos board with male and female headers soldered.

The Hypnos board requires two sets of headers. Solder male headers pointing down onto the Feather Rail and female headers facing up onto Sensor/Power Rail of the Hypnos board (Fig. 4A). Fig. 4B shows a completed Hypnos Board. Insert a CR1220 coin cell into the slot on the bottom side of the Hypnos board.

##### I^2^C multiplexer

**Fig. 5:**
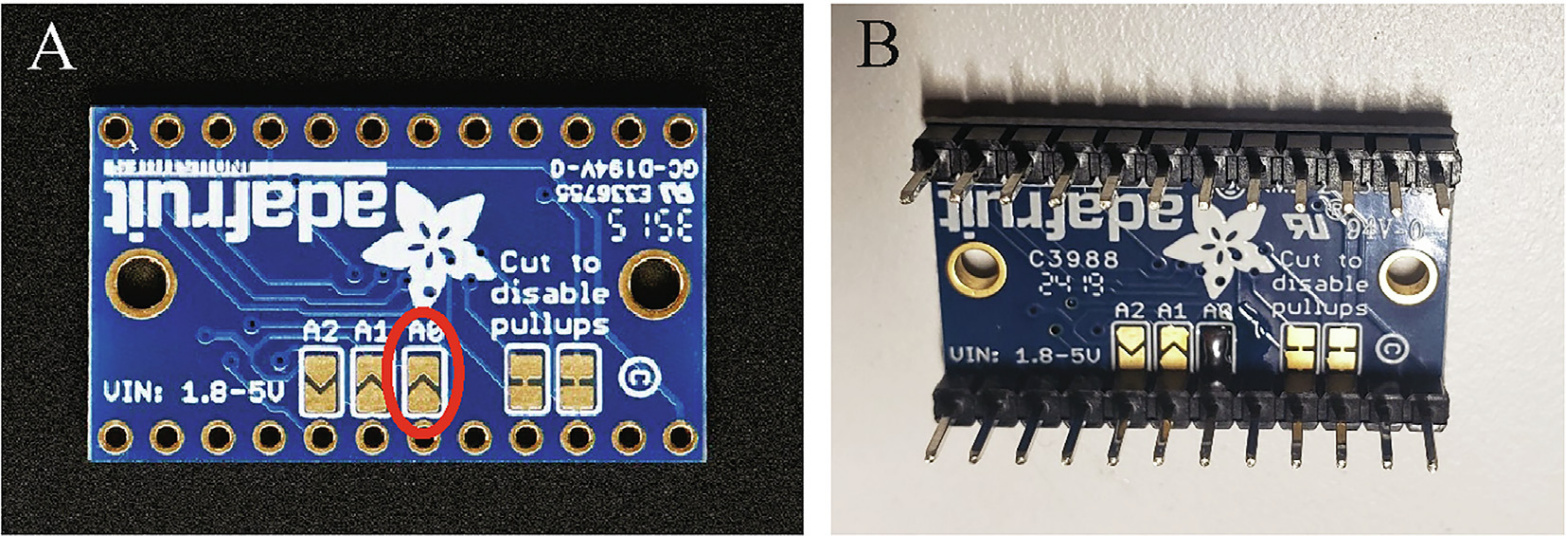
(A) Unsoldered TCA9548A breakout with red circle identifying A0 solder jumper. (B) TCA9548A breakout with male header pins and I^2^C jumper soldered. (For interpretation of the references to color in this figure legend, the reader is referred to the web version of this article.)

The I^2^C multiplexer requires one set of headers (included) and a jumper. Solder the A0 I^2^C address jumper (circled in red on Fig. 5A) on the TCA9548A breakout and solder male headers to the bottom of the TCA9548A breakout. Fig. 5B shows the TCA9548A breakout

**Fig. 6:**
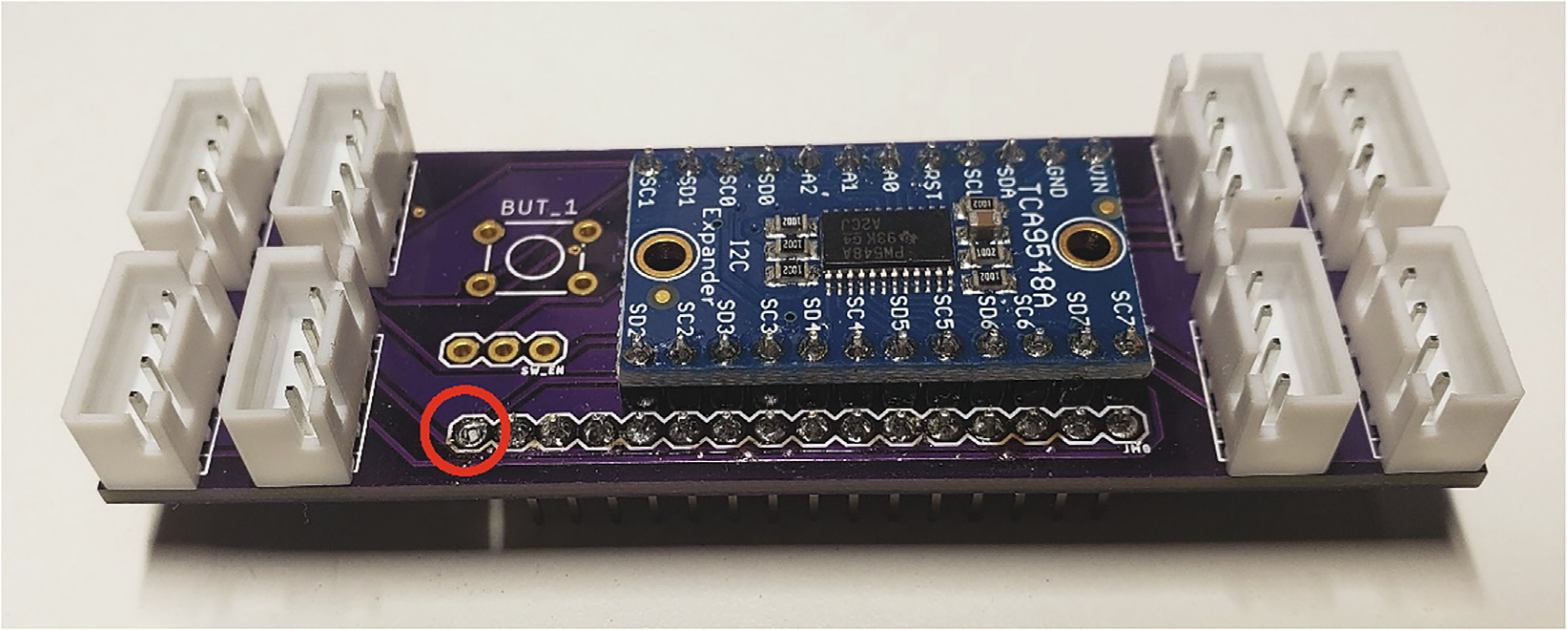
Multiplexer board with JST XH-4, headers and TCA9548A breakout.

The I2C multiplexer board requires one set of headers, the TCA9548A breakout board, and 8 JST connectors. Set the TCA9548A breakout onto the I^2^C multiplexer board and solder the male header pins to the board. Trim the headers from the TCA9548A breakout sticking down past the I^2^C multiplexer board. Solder 8 JST XH-4 connectors to the multiplexer board, making sure to orient the side of the connecter with notches towards the center of the board. Solder male headers on the bottom of the multiplexer board excluding the reset pin as shown by the red circle in Fig. 6. The switch and button footprints on the multiplexer board are left unpopulated because they are not necessary for this project.

##### Feather doubler, SDI-12, tipping bucket, and accelerometer wiring

**Fig. 7:**
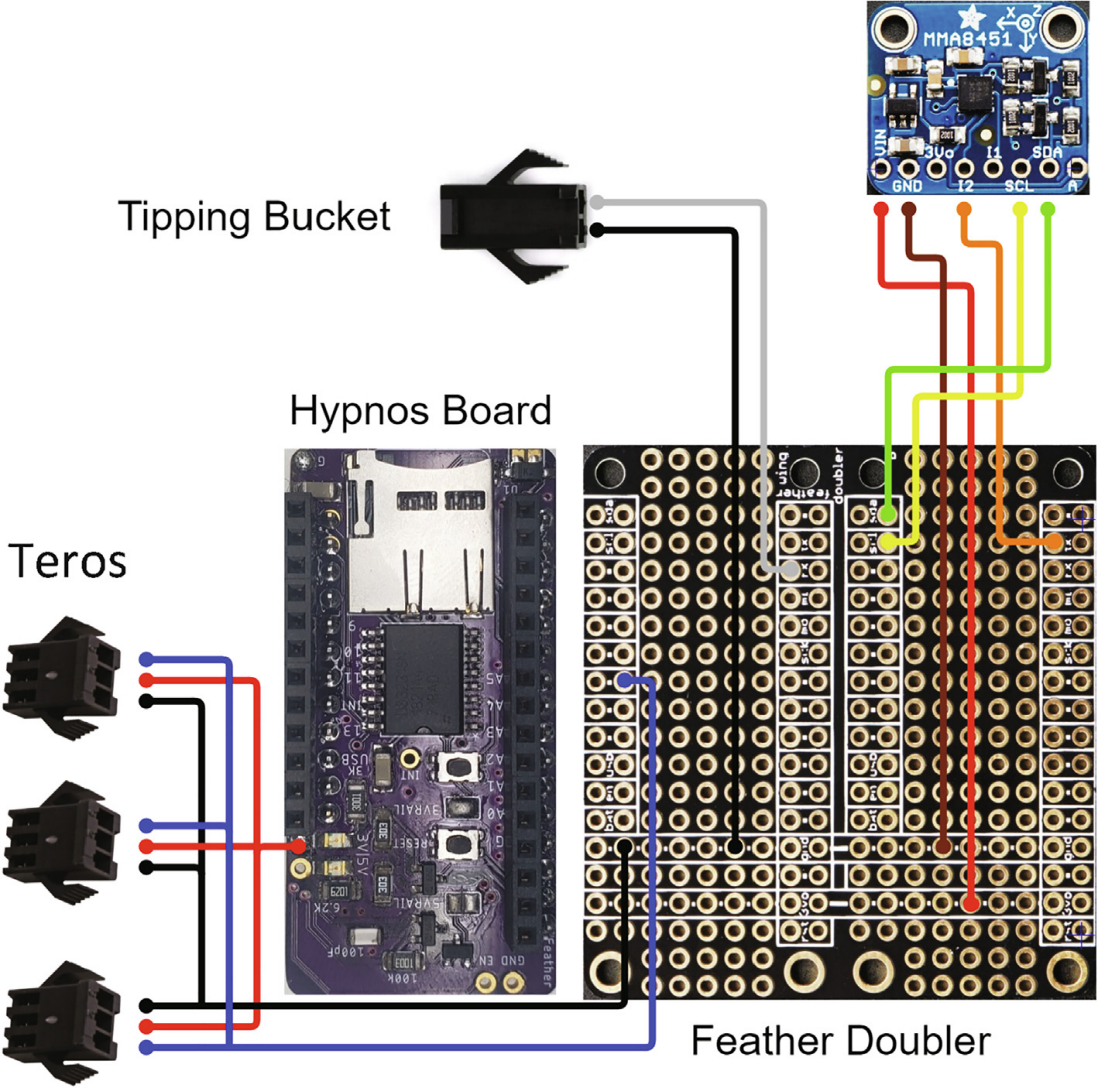
Wiring diagram for MMA8451 and tipping bucket and SDI-12 Teros 11 connectors to Hypnos (left, top side up) and Feather Doubler (right, top side up).

**Fig. 8:**
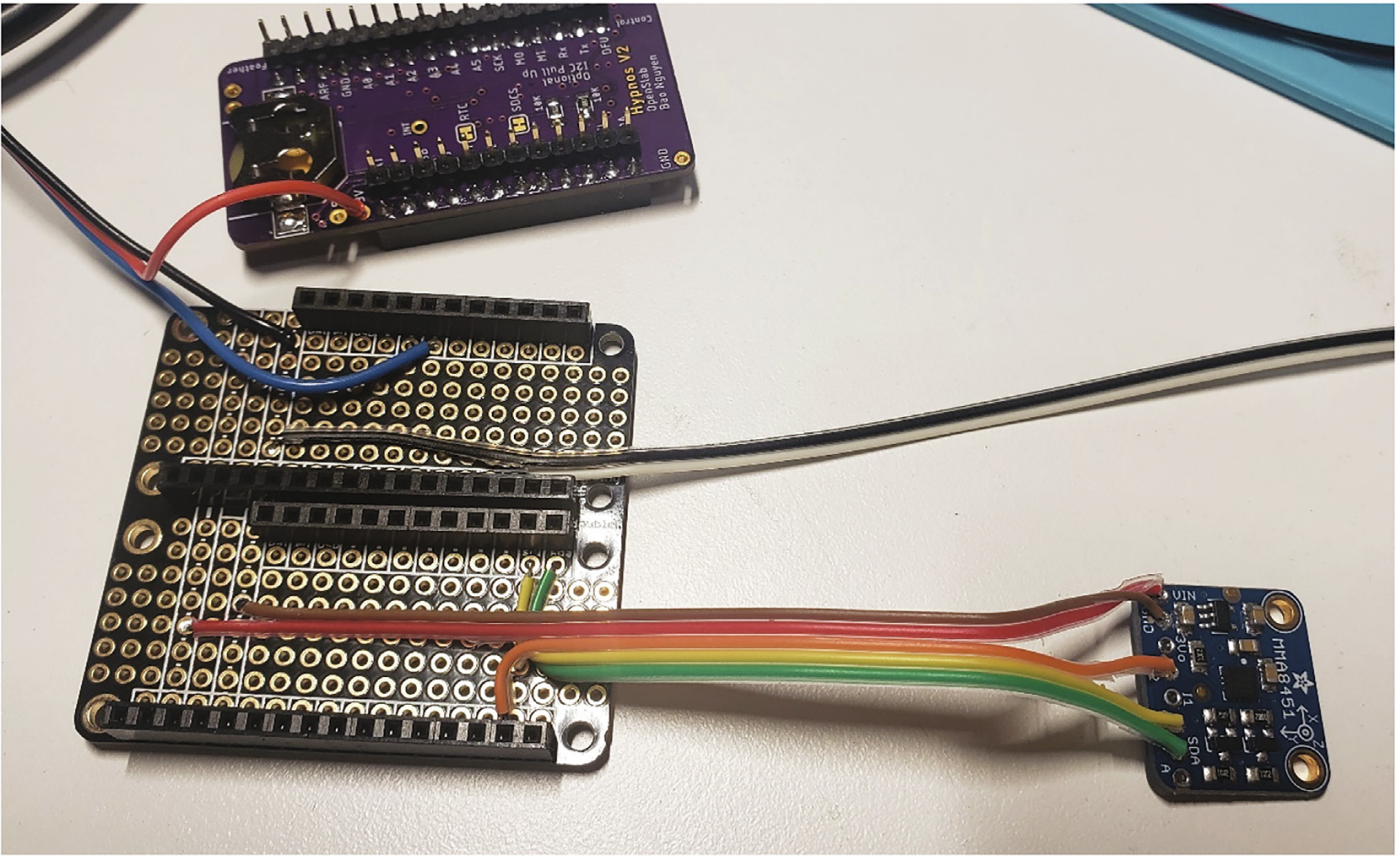
Feather Doubler (bottom left, top side up) and Hypnos (top, bottom side up) with female headers and MMA8451 (bottom right), SDI-12, and tipping bucket wires soldered.

Three sets of wires are soldered to the Feather Doubler and one also to the Hypnos board. Solder one end of a short length (about 6 in.) of ribbon cable to the Feather Doubler and crimp a female JST SM-2 connector onto the other end (Fig. 7, Table 2). This connector is for the tipping bucket and is shown with black and white wires. Solder another approximately 6 in. long section of ribbon cable to the Feather Doubler for the Teros sensors (black, red, and blue) and crimp 3 female JST SM-3 connectors to the other end. Using a section of ribbon cable approximately 4 in. long, solder the MMA8451 accelerometer (red, brown, orange, yellow, and green) on to the Feather Doubler and Hypnos as shown in the wiring diagram (Fig. 7, Table 2). Solder 1 set of 12-pin and 16-pin female headers on top of the outer rails of each side of the Feather Doubler (Fig. 8). Fig. 8 shows the completed Feather Doubler with the accelerometer, SDI-12, and tipping bucket wires soldered.

**Table 2:** Connections for the tipping bucket and SDI-12 wires to the Feather Doubler board and Hypnos board.

**Sensor**	**Sensor wire color**	**Board pin**
**Tipping bucket**	Black	Feather Doubler: GND
**Tipping bucket**	White	Feather Doubler: Pin 0
**MMA8451 Accelerometer**	GND (brown)	Feather Doubler: GND
**MMA8451 Accelerometer**	3.3 V (red)	Feather Doubler: 3.3 V
**MMA8451 Accelerometer**	I2 (orange)	Feather Doubler: Pin 1
**MMA8451 Accelerometer**	SCL (yellow)	Feather Doubler: SCL (Pin 21)
**MMA8451 Accelerometer**	SDA (green)	Feather Doubler: SDA (Pin 20)
**SDI-12**	Black	Feather Doubler: GND
**SDI-12**	Red	Hypnos: 3.3 V rail
**SDI-12**	Blue	Feather Doubler: Pin 11

**Fig. 9:**
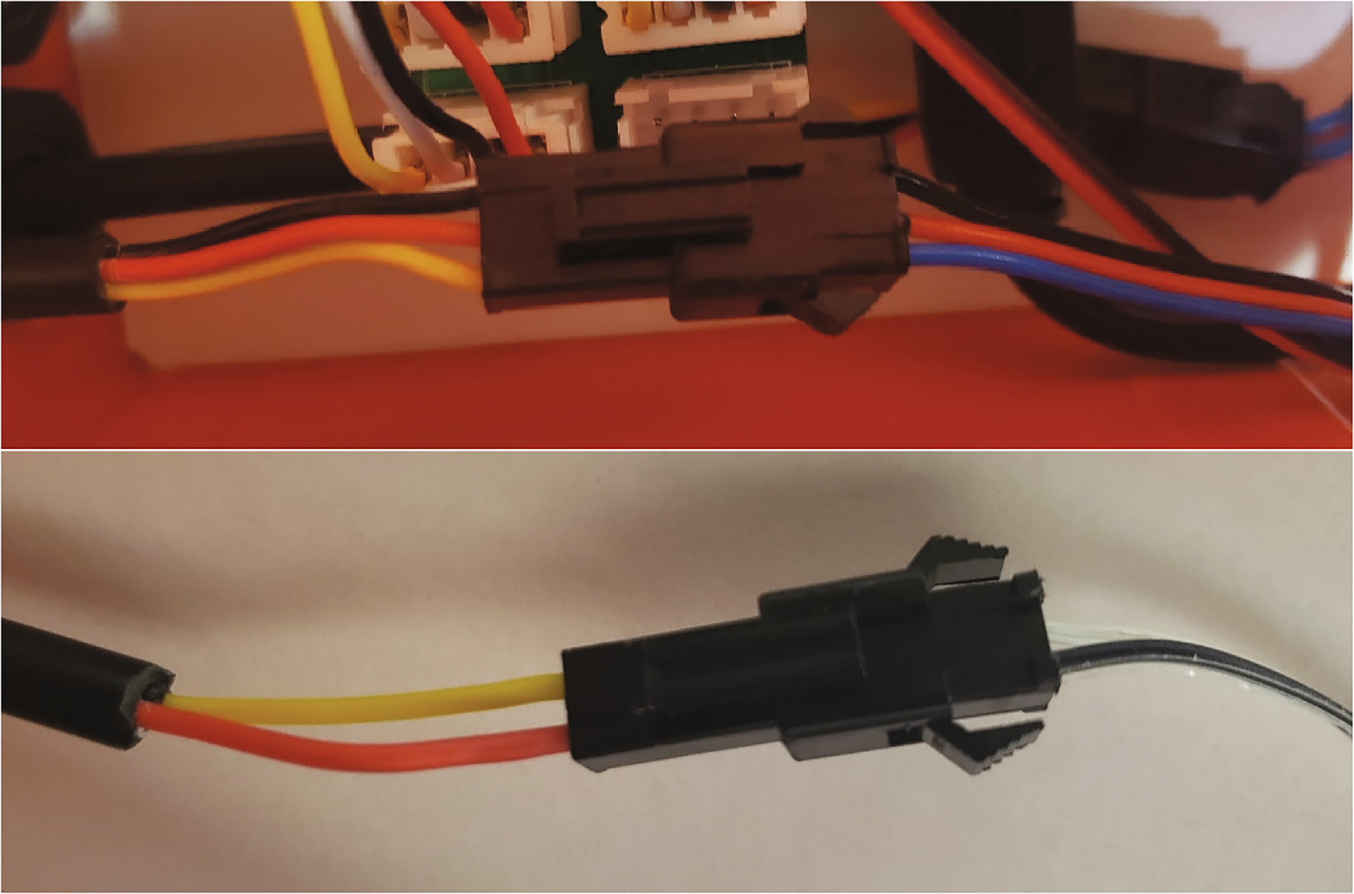
SDI-12 and tipping bucket wires with JST SM connectors.

To allow for sensor removal separate from the data logger, the ends of the polarized cables inside the pelican case need to have connectors attached. First, thread the polarized cables through the cord grips. Crimp a male JST SM-3 connector to the inside end of the pelican case of the three polarized cables connected to the Teros sensors (red, black, yellow wires, Fig. 9, top) and a male JST SM-2 connector to the inside end of the tipping bucket polarized cable wires (yellow and red wires, Fig. 9, bottom).

**Fig. 10:**
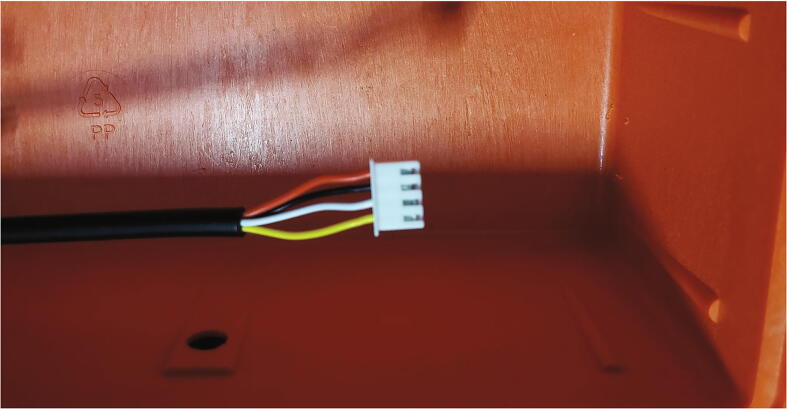
JST XH-4 connector crimped to the end of the polarized cable inside the pelican case.

Crimp female JST XH-4 connectors to the inside end of the remaining six polarized cables as shown in Fig. 10.

##### STEMMA sensors

**Fig. 11:**
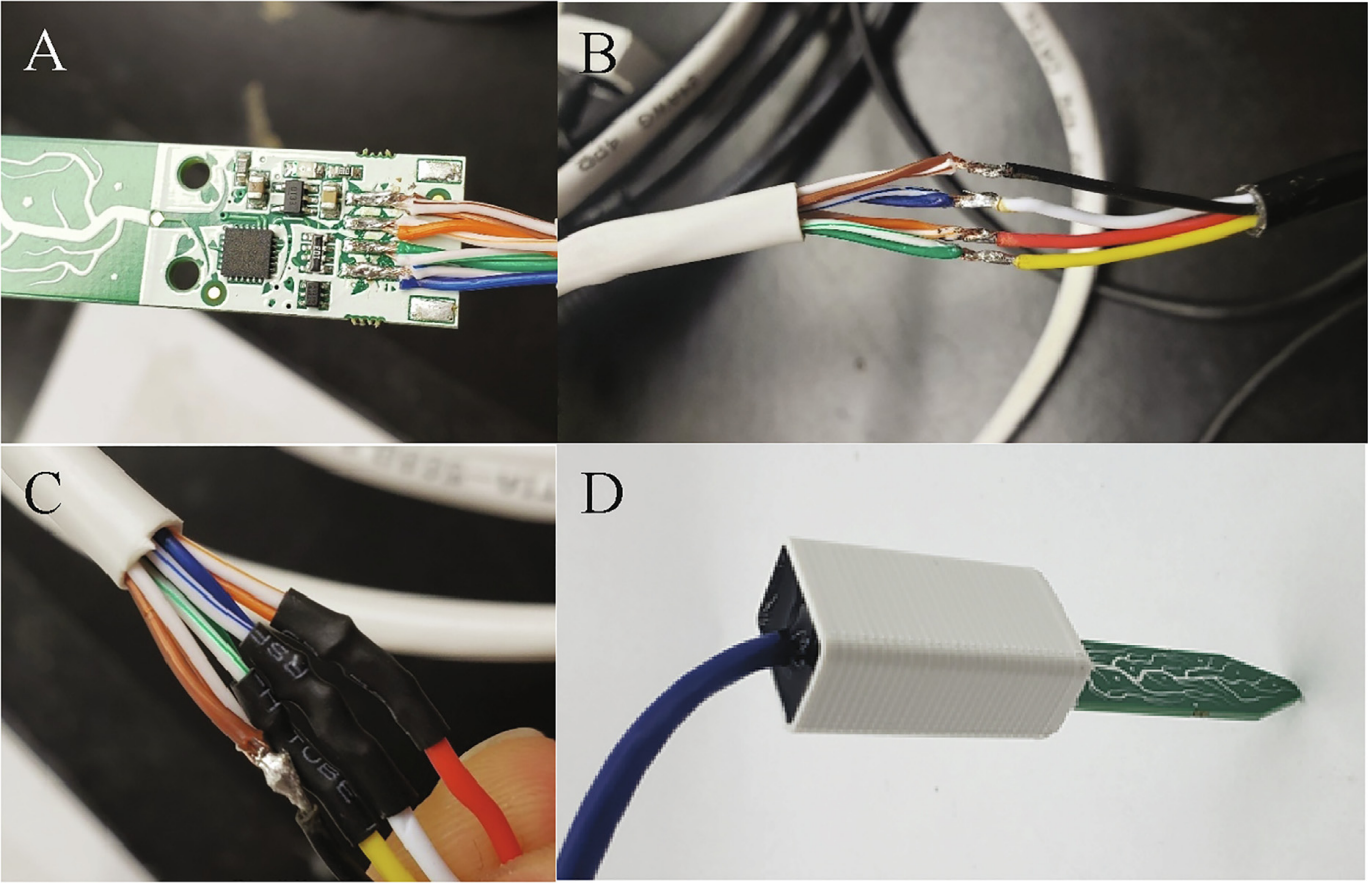
A) STEMMA sensor with black connector removed and ethernet cable soldered. B) Ethernet cable soldered to waterproof cable. C) Ethernet cable to waterproof cable connection with heat shrink tubing over solder joints for 3 wires. D) Potted STEMMA sensor.

The stock cable for the STEMMA sensors needs to be replaced with a longer and more robust option so that the I^2^C signal can travel along the required cable length, and the sensor electronics need to be protected. Using a soldering iron, de-solder the black connectors from the STEMMA sensors. Then, cut the connectors off of the ethernet cable and cut three 10ft sections of ethernet cable. On both ends of the ethernet cable, strip back the outer jacket and individual wire jackets. Solder one end of each 10ft section of ethernet cable to a STEMMA sensor. Each pin on the sensor is soldered to one twisted pair of wires from the ethernet cable. (Fig. 11A, Table 3).

**Table 3:** Wiring connections for STEMMA sensor to ethernet cable.

**Wire color**	**STEMMA pin**
Brown	GND
Orange	3.3 V
Green	SDA
Blue	SCL

Slide a 1/2″ long piece of 1/16″ heat shrink onto each of the four wires of the polarized connector and an approximately 4″ long piece of 5/16″ heat shrink onto the unused end of the ethernet cable. Solder each twisted pair from ethernet cable to a wire on the polarized connector following Table 4 and Fig. 11B. Use a heat gun to shrink the heat shrink over the individual wire joints (Fig. 11C). Slide the 5/16″ heat shrink over the bundle of wire joints, and use the heat gun to shrink the heat shrink over the joint between the ethernet cable and the polarized cable to insulate and protect the connection. Push the STEMMA sensors through the slot in the STEMMA Case so that the case is covering the SMD components on the sensor. Then fill the cavity with potting compound until the potting compound is flush with the top of the cavity (Fig. 11D).

**Table 4:** Wiring connections for ethernet cable to polarized waterproof connector.

**Ethernet twisted pair color**	**Polarized connector wire color**
Brown	Black
Orange	Red
Green	Yellow
Blue	White

##### Pressure and humidity sensors

**Fig. 12:**
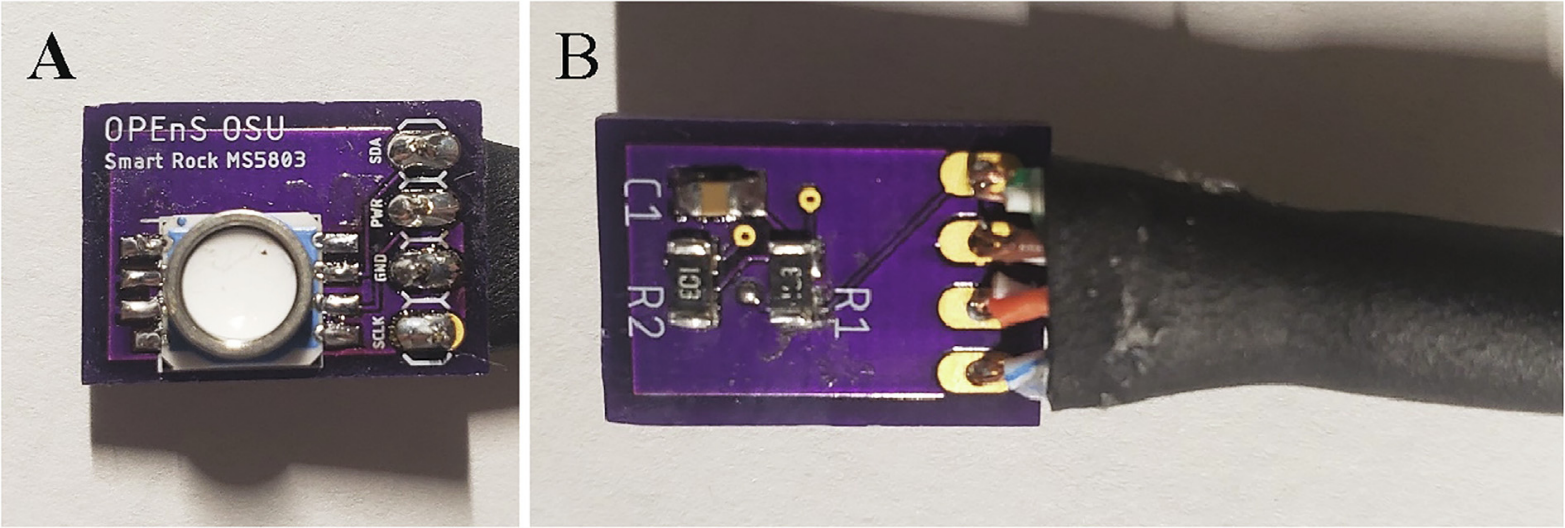
A) MS5803 soldered to the top of the PCB. B) Bottom of MS5803 PCB with SMD components and cable soldered.

To attach all the components to the pressure sensor breakout boards, start by orienting each pressure sensor breakout board with the text on the PCB facing up (Fig. 12A). Align the blue dot on each pressure sensor to the top left corner of the pad for the pressure sensor on each breakout board (Fig. 12A) Solder the eight pads of each MS5803 pressure sensor to a pressure sensor breakout board. Flip over each breakout board and solder two 10kΩ resistors (R1 and R2) and one 100nF capacitor (C1) to the back of each breakout board (Fig. 12B).

The pressure and humidity sensors will be connected to the microprocessor with polarized connectors, and, for one pressure sensor, ethernet cable as well. One pressure sensor and the humidity sensor will be outside and directly below the pelican case, and the other pressure sensor will be in the well point. To protect the joint after soldering, add a 1″ long piece of 5/16″ heat shrink over two polarized connector cables. The polarized connector cable can optionally be shortened. Solder the four wires from one piece of a polarized connector directly to one MS5803 pressure sensor (Table 5) and one piece of another polarized connector directly to the SHT31D humidity sensor. Slide the heat shrink to be flush with the edge of the PCB for one pressure sensor and the humidity sensor (Fig. 12, Fig. 14) and use a heat gun to shrink the heat shrink. For the second MS5803 pressure sensor that will be in the well point, cut off a 5 ft section of ethernet cable, use heat shrink to protect the solder joints as described in Section 5.1.1.5 and Fig. 11, and solder one end of the ethernet cable to the second MS5803 pressure sensor and one end to the polarized connector following Table 5.

**Table 5:** Wiring connections for MS5803 to pressure sensor.

**Polarized connector wire color**	**MS5803 Breakout pin**
Red	VIN
Black	GND
White	SCL
Yellow	SDA

**Fig. 13:**
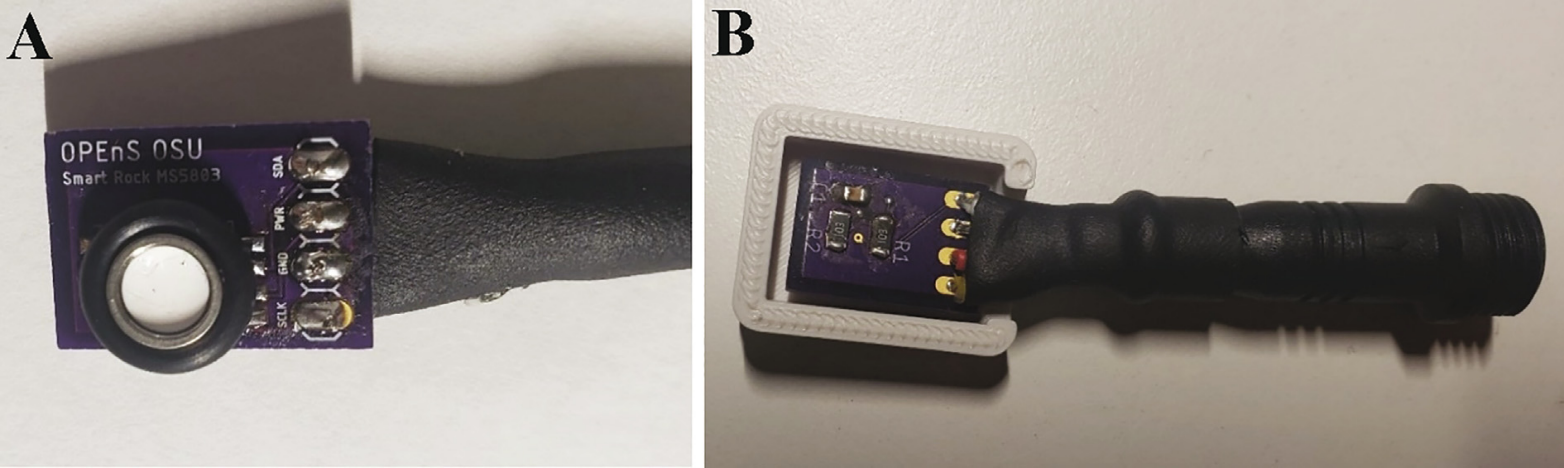
A) MS5803 sensor with O-ring. B) MS5803 with its case.

The 3D printed cases, o-rings, and epoxy are used to protect the electronics of the pressure sensors and humidty sensors. Fit a 008O-ring around each MS5803 pressure sensor (Fig. 13A). Each pressure sensor can then be pressed into a MS5803 case (Fig. 13B).

**Fig. 14:**
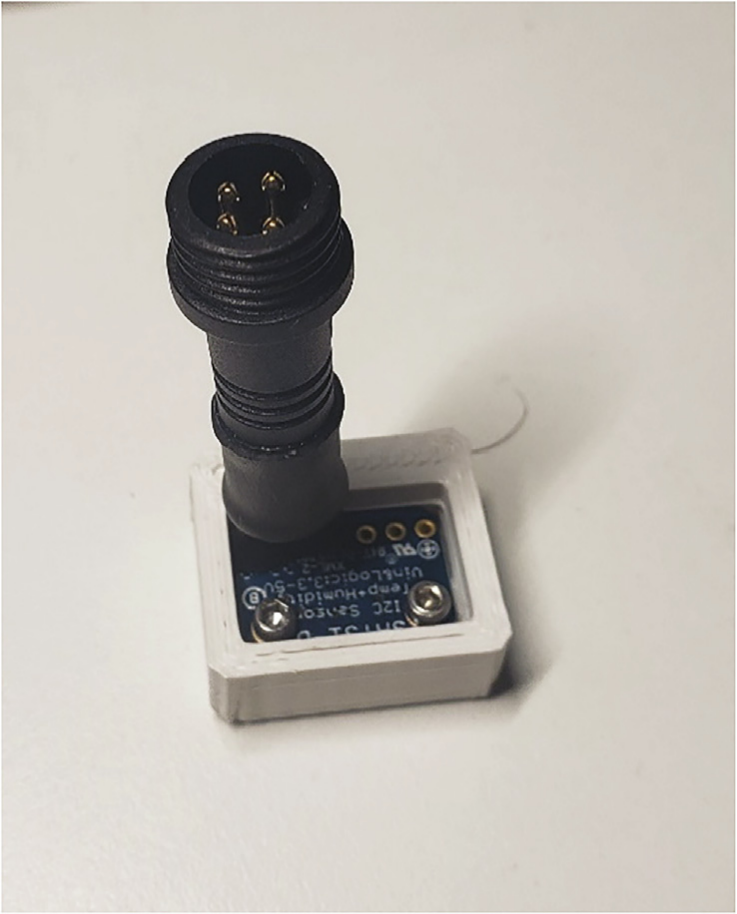
SHT31D screwed into its case.

Attach the SHT31D humidity sensor to the SHT31D Case using 2 M2-0.4 × 2 mm screws (Fig. 14). Fill the SHT31D and both MS5803 cases with potting compound to the top of the 3D printed cases.

##### JST parallel PCB

**Fig. 15:**
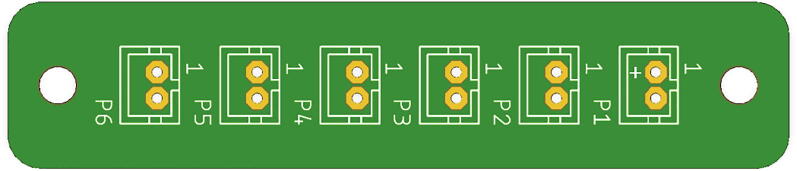
JST Parallel PCB without connectors or extension wire.

The JST Parallel PCB provides connection points for five batteries as input, and a single connection to the Feather M0 for output. Solder five JST SM connectors to the P2-P6 footprints on the JST Parallel PCB for the batteries. Orient the notch in each of the connectors to follow the silkscreen (Fig. 15). Solder a JST SM extension to the P1 footprint on the PCB, making sure the red wire is connected to the positive terminal and the black wire is connected to the negative terminal. On Fig. 15, the positive terminal is the top pad. Connect the JST SM extension to the battery connector on the Feather M0.

##### Pelican case modifications

**Fig. 16:**
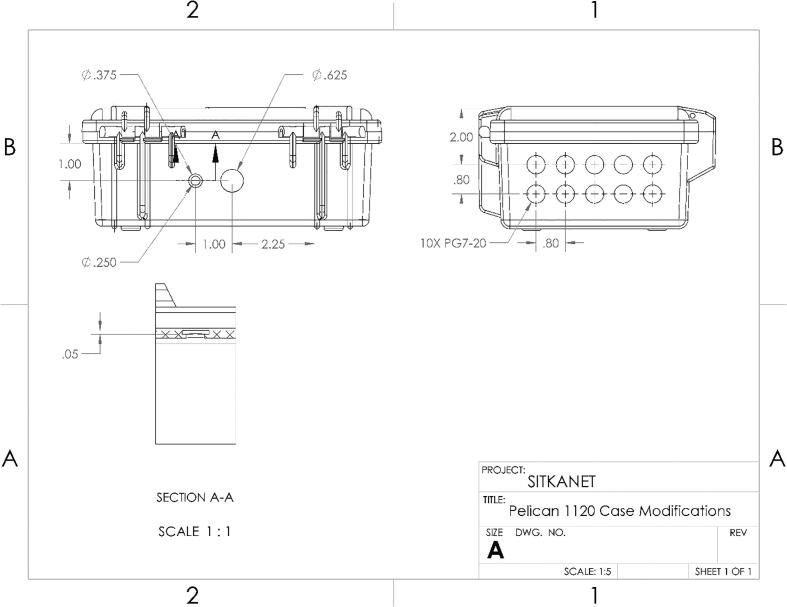
CAD drawing of modifications to Pelican case.

To allow for individual, waterproof pass through of sensor cables, antenna cables, and USB access, twelve holes need to be drilled and filled. Remove and discard the foam from the bottom of the pelican case. For the sensor cables, drill ten 7/16″ holes and tap the holes with PG7-20 threads into the bottom of the pelican case according to Fig. 16. The holes are arranged in two rows of five, spaced 0.8 in. apart. The holes are centered horizontally on the bottom face of the case, and the center of the top holes 2 in. from the top of the lid. Thread 10 PG7 cable grips into the pelican case.

For the USB port, drill a 5/8-inch diameter hole centered horizontally and vertically on the left face of the case (Fig. 16 2B). For the antenna, drill a 1/4-inch diameter hole with a 3/8 in. diameter counterbore 0.1-inch deep 1-inch center-to-center to the left of the 5/8″ hole (Fig. 16).

##### Final assembly

The Case Insert provides the mounting surface for all the electronics in the case. Using a soldering iron heated to approximately 200 °C, press 6 M2 heat-set threaded inserts into the 6 holes in the Case Insert. Put the Case Insert into the Pelican case oriented with the microSD extension closest to the antenna hole and ensure it is a snug fit. The Case Insert is designed to be a friction fit in the case, but glue can be added to make a permanent connection.

**Fig. 17:**
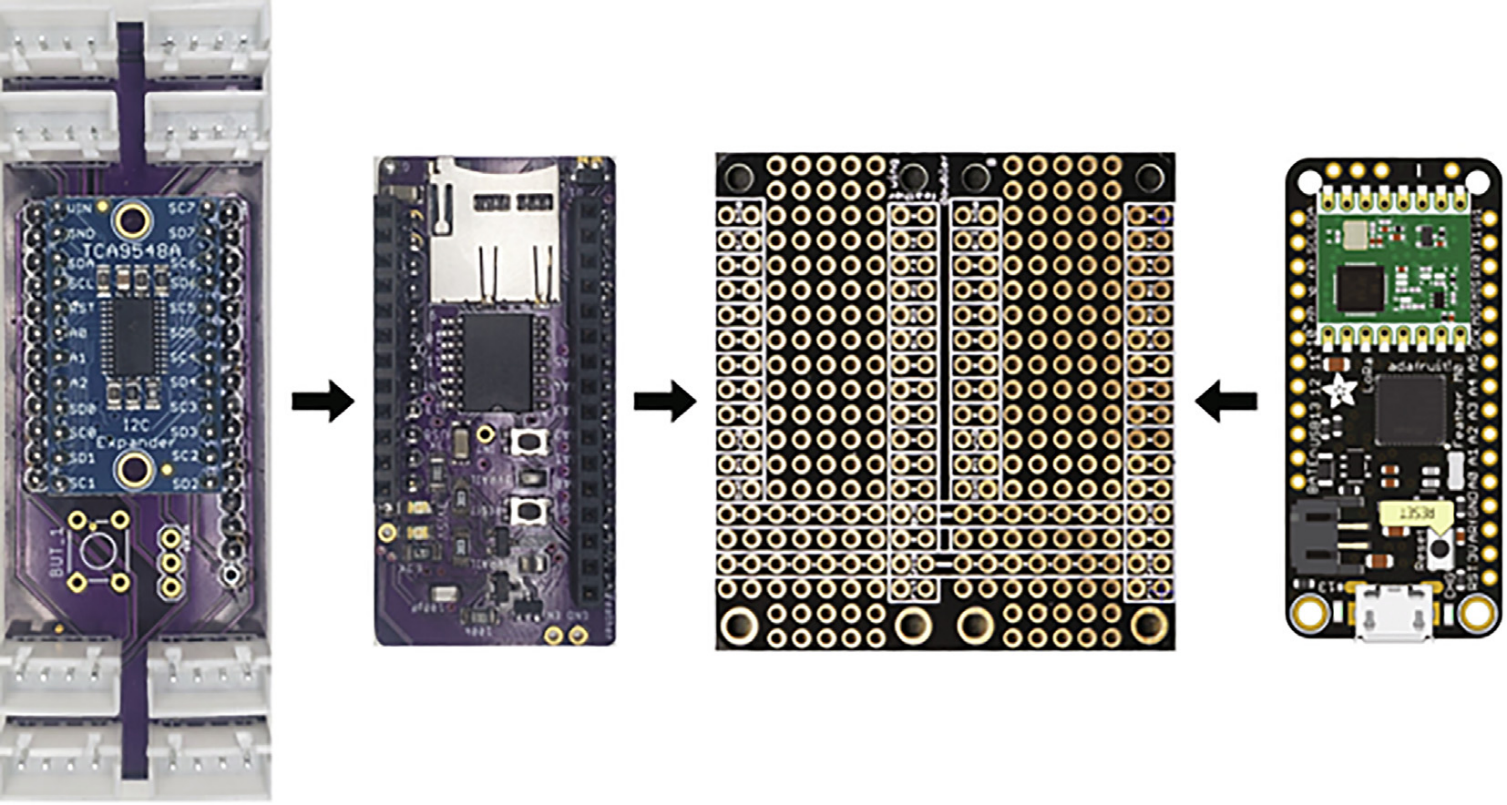
Arrangement of the I^2^C multiplexer, Hypnos, Feather Doubler, and Feather M0.

Using the M2 screws, secure the Feather Doubler, MMA8451 Accelerometer, and JST parallel PCB to the Case Insert. As shown in Fig. 17, stack the Feather M0 by itself on the right side of the Feather Doubler, the Hypnos board first on the left side of the Feather Doubler, and the I^2^C multiplexer on top of the Hypnos board.

**Fig. 18:**
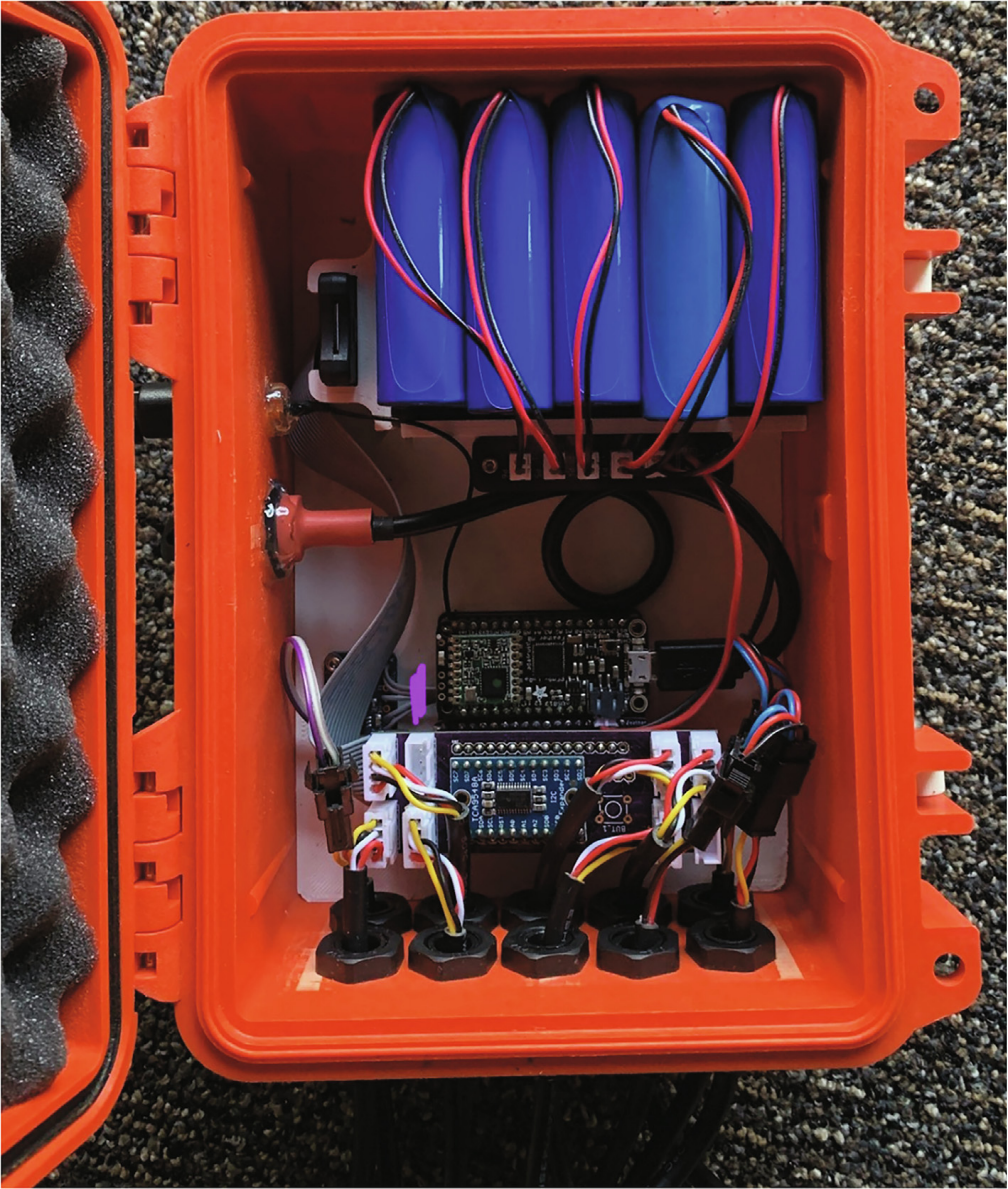
Inside view of a completed node control box.

Insert the microSD card into the extension and slide it vertically down into the corresponding slot on the Case Insert. Connect the other end to the microSD card socket on the Hypnos Board. Insert the 6600 mAh batteries into the Case Insert with the wires coming out on the top side at the top of the case, but do not plug in the batteries until ready to operate (Fig. 18). Plug in the USB extension to the Feather M0. Mount the USB extension, USB extension cap, and antenna adapter cable to the wall of the Pelican case using the nuts supplied with each connector and apply hot glue to the interface of the connector and the inside wall of the pelican case.

**Fig. 19:**
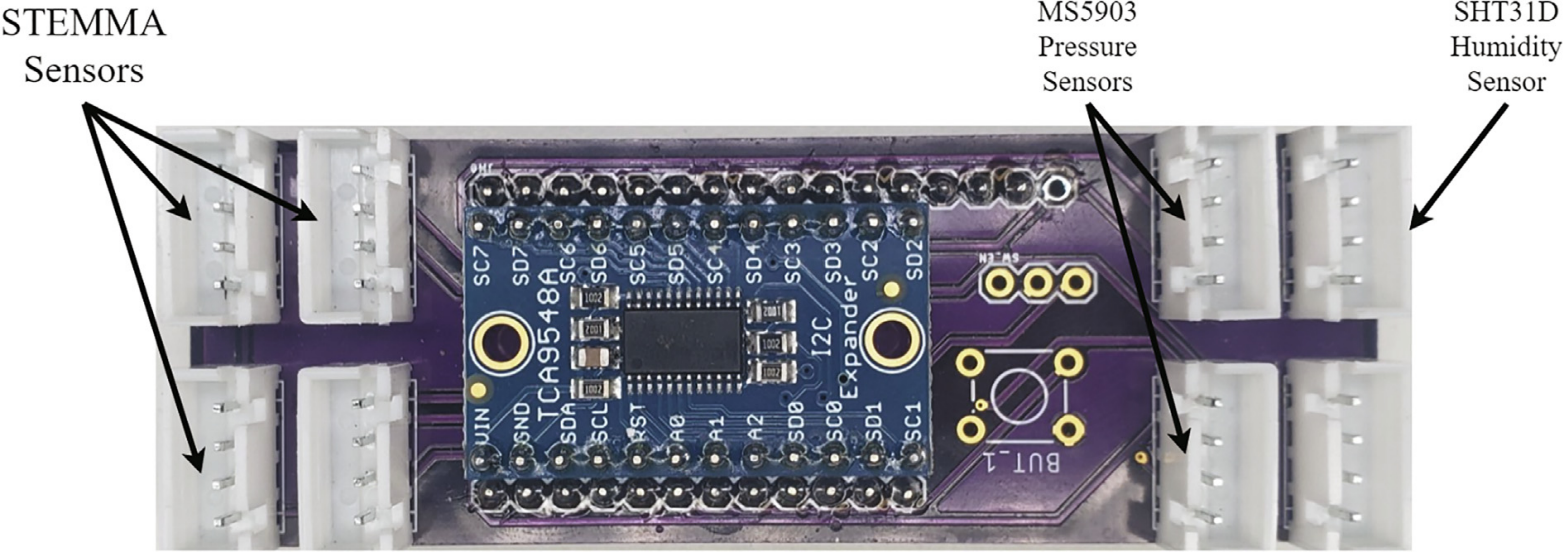
I^2^C Multiplexer with labels showing the locations where sensors should be plugged into.

Thread the waterproof cables through the cord grips and connect them to their respective JST connectors on the I^2^C multiplexer (Fig. 19). Follow these instructions to install LOOM on your computer. Then use the Arduino IDE to compile and upload the Node.ino code to the Feather M0.

**Fig. 20:**
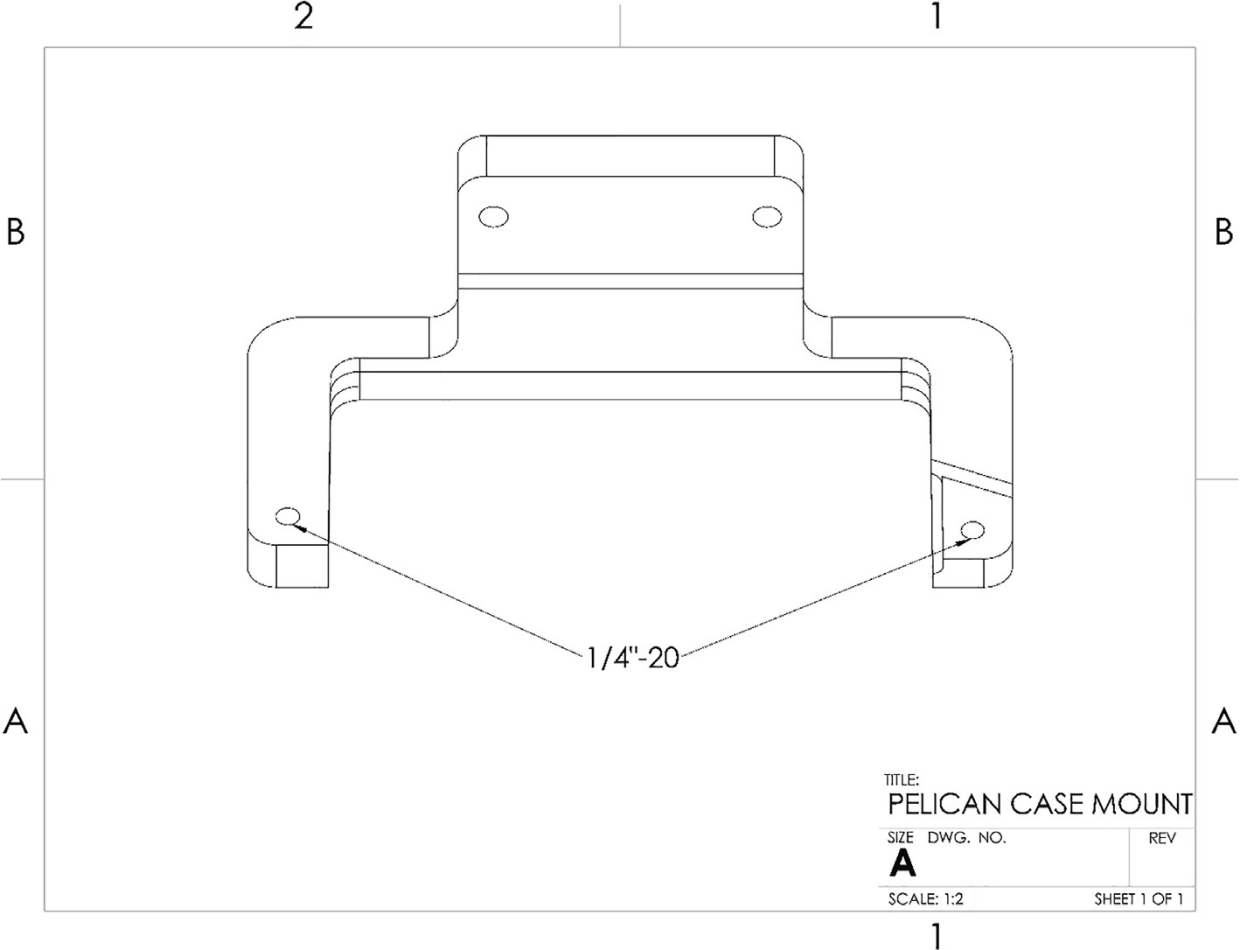
CAD drawing showing location of tapped holes on Pelican Case Mount Top and Bottom.

Push the pins holding the pelican case handle out. Tap the labeled holes (Fig. 20) on the Pelican Case Mount Top and Pelican Case Mount Bottom to 1/4″-20. The Pelican Case Mount Top and Bottom are a friction fit around the back and sides of the Pelican Case. Align the mounts with the ridges on the case and make sure the mounts are fully seated against the outer wall. Secure the mounts to the case using the 1/4–20 bolts through the handle pin holes.

**Fig. 21:**
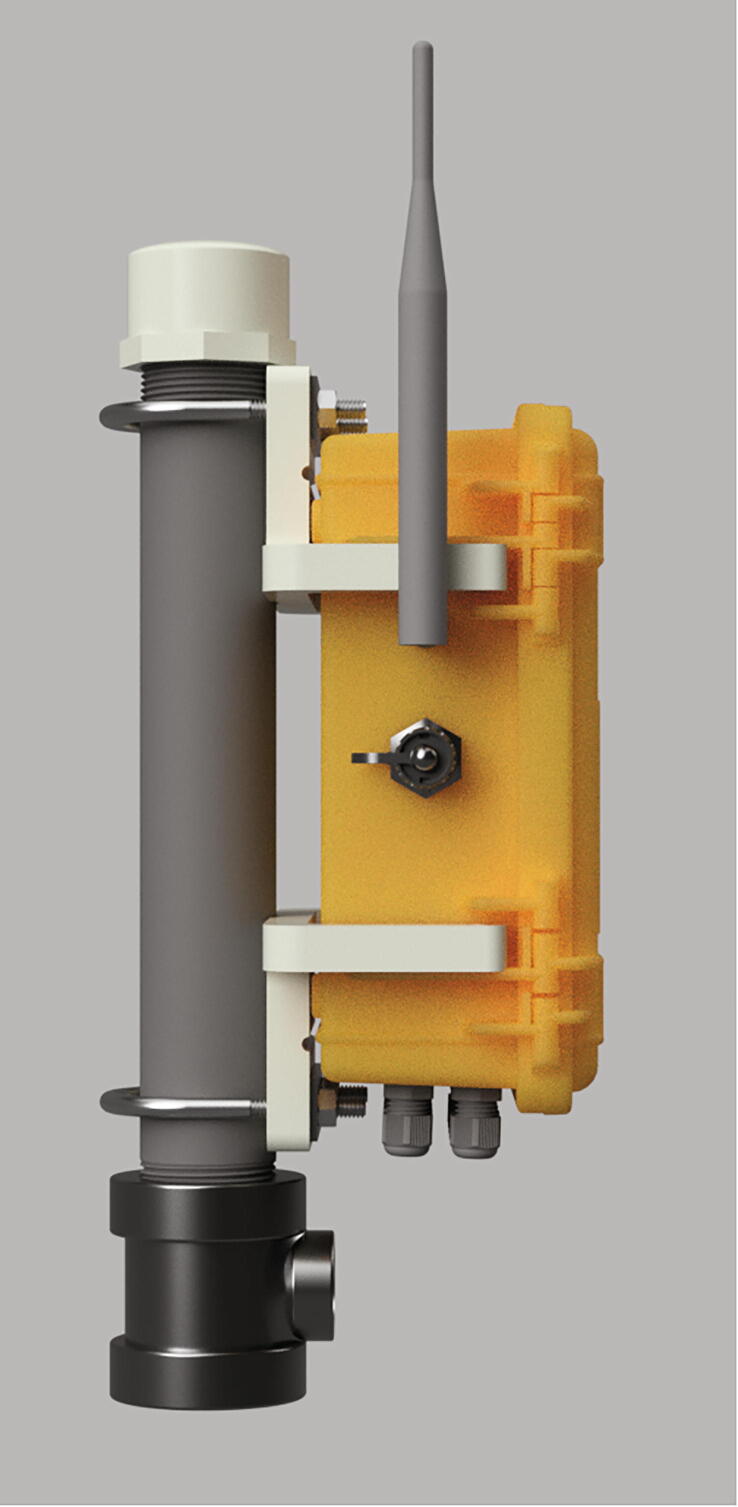
Node attached to PVC pipe, cap and tee.

Screw the PVC Cap, PVC Pipe and Pipe Tee together. Then use the U-bolts to attach the Pelican case to the PVC pipe (Fig. 21). Tighten the antenna onto the antenna adapter cable.

### Hub build instructions

After 3D printing the Hub Case, remove any support material. Use a soldering iron heated to approximately 200 °C to press the M3 threaded inserts into the 4 holes at the corners of the case. From the outside, push the DC barrel jack into its corresponding hole in the Hub Case and attach and tighten the nut on the inside of the case. Attach the antenna adapter cable to the Hub Case by pressing it from the inside into the matching hole in the case and attaching and tightening the nut on the outside of the case.

**Fig. 22:**
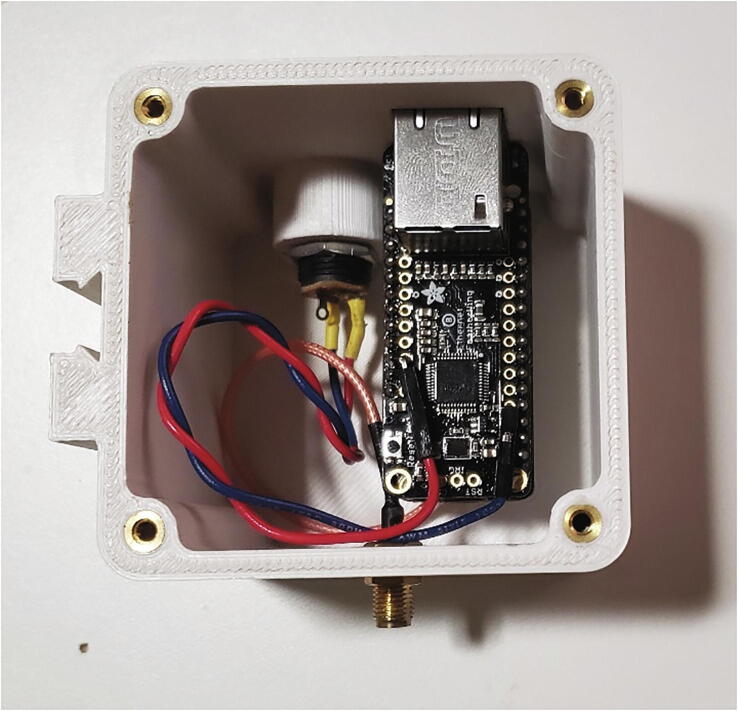
Completed hub without lid.

Solder female headers on the top of the Feather M0 LoRa and male headers protruding from the bottom of the Ethernet Featherwing so that the Featherwing can stack on top of the Feather. Solder the u.Fl connector to the Feather M0, connect the antenna adapter cable, and add hot glue to the connection (Section 5.1.1.1, Fig. 3). Solder and heat shrink wires from the positive terminal and negative terminal of the DC barrel jack to the USB and GND pins on the Ethernet Featherwing. Fig. 22 shows a completed hub without the lid and antenna. Use the M3 screws to attach the Hub Lid to the Hub Case. Connect the antenna to the hub through the RF cable. Follow these instructions to configure the Google Sheets spreadsheet where data will be uploaded. Then use the Arduino IDE to compile and upload the Hub.ino code to the Feather M0.

## Operation instructions

### Node installation

Sensor sites were placed in areas where landslides were likely to initiate in areas of steep slope angle and convergent topography (e.g. [22], [23]). Convergent topography accumulates both groundwater and loose sediment. Identification of potential sites was based on remote topographic imagery, field observation, and slope stability analysis using the SHALSTAB shallow landslide model [24]. In Sitka, all six sites are located within topographic depressions or debris channels on very steep slopes (>35°) with comparable geomorphic characteristics (elevation, forest type, soil type).

Once a node site was selected, the well point was driven into the soil using a driving cap and a small sledgehammer, leaving the threads exposed above the soil. The driving cap and sledge method was chosen to balance weight and bulk of tools and ease of installation. Next, a soil pit approximately 1 m deep by 25 cm wide was dug near the well pipe. The soil moisture sensors were installed into the side of the soil pit spaced evenly along the depth of the soil pit. The maximum distance between the well pipe and soil pit is limited, in combination with the maximum desired sensing depth, to the 5 ft length of the STEMMA sensor cables. The soil pit was then filled back in. The well pressure sensor was inserted into the bottom of the well. Next, the control box assembly was attached to the well pipe and the tipping bucket was mounted on a level surface in an area with a canopy gap within the 40′ cable length. All sensors were securely plugged into their respective ports (Fig. 19). Finally, the JST connectors of each of the five batteries were plugged into ports P2 through P6 on the JST Parallel PCB (Fig. 18) and successful data transmissions were confirmed by checking the Google Sheet.

### Hub installation

Plug an ethernet cable with internet access to the hub. Connect the Yagi antenna to the hub using the RF cable. Then plug in the power supply and check for successful data reception and upload to the spreadsheet. Positioning the Yagi antenna to optimize line-of-sight to all node antennas is advisable for maximum performance.

## Validation and characterization

**Fig. 23:**
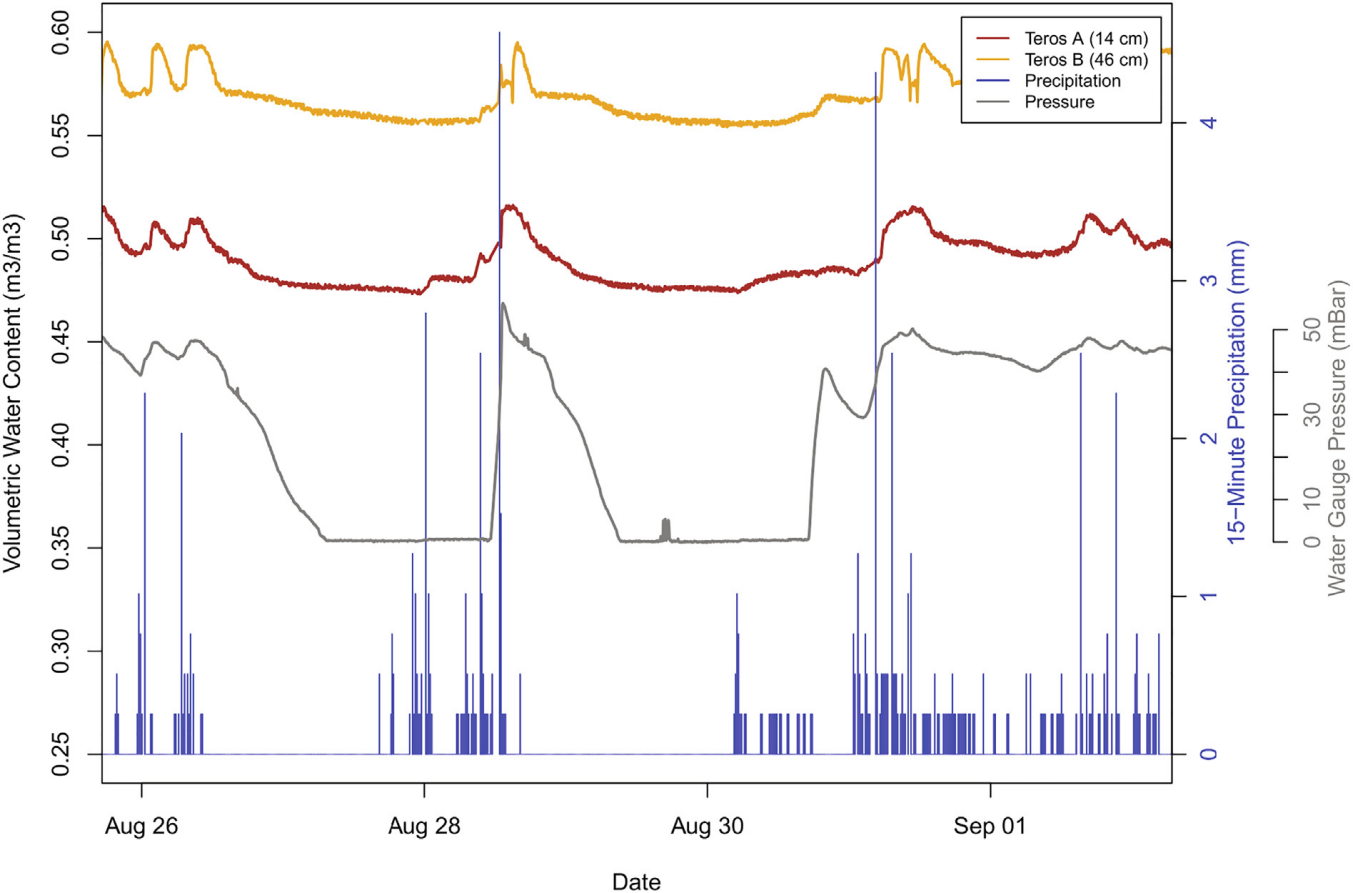
Example data stream from Harbor Mountain sensor deployed in Sitka in summer 2020, showing the relationship between rainfall and hydrologic response.

**Fig. 24:**
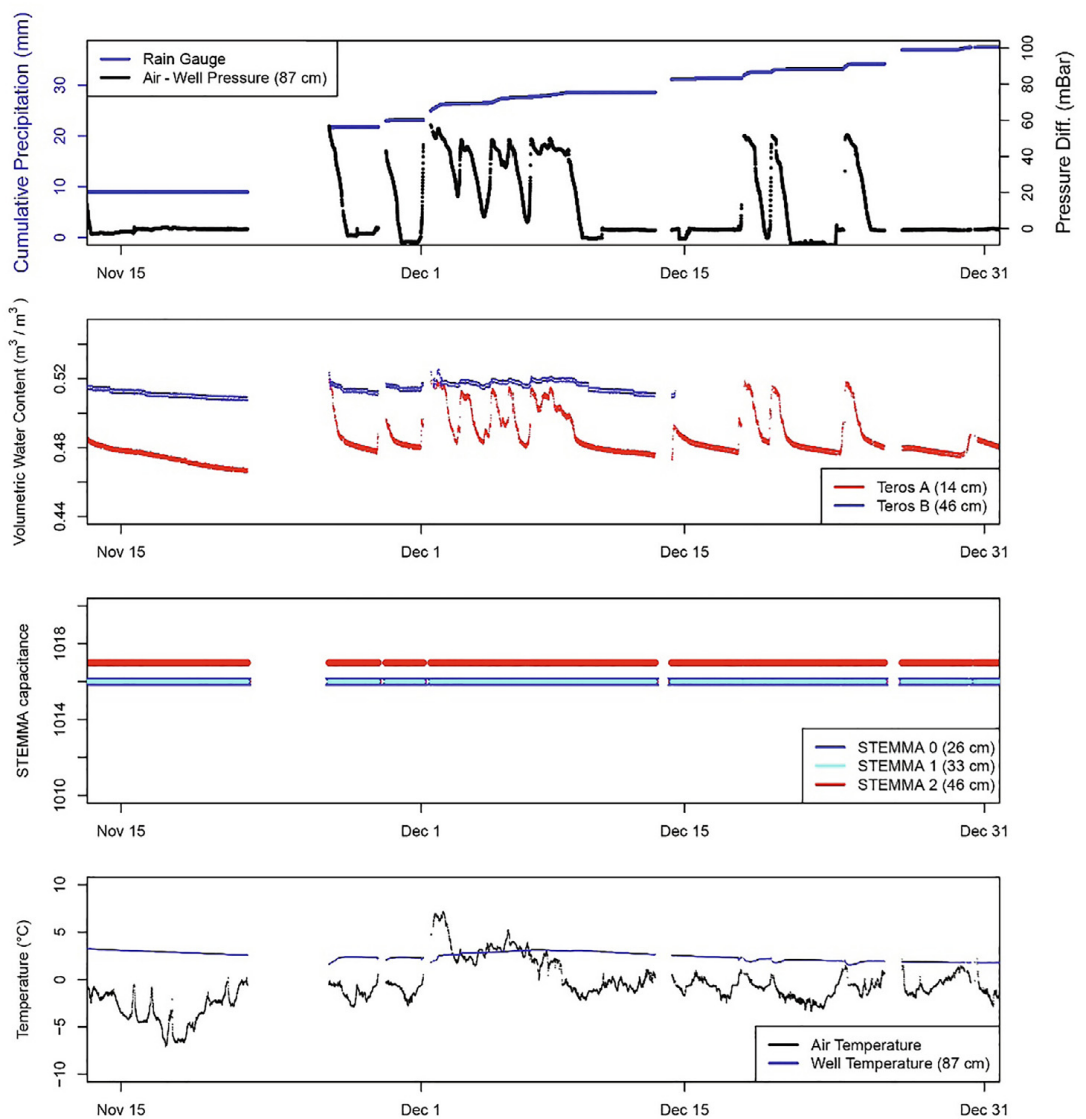
Example of the multiple data types transmitted to the receiving hub at the same Harbor Mountain Station in November to December 2020. The third Teros soil moisture sensor (Teros C) reported 0% saturation for the entire period of record shown here. The three STEMMA sensors reported maximum values for the entire period of record (1016 or 1017) without any variance in response to rainfall. Notable gaps in data when LoRa transmissions were not successful occurred during periods of heavy rainfall, such as the storm at the end of November.

Successful data transmission from three susceptible hillslopes (Fig. 23) demonstrated that cost-effective soil moisture monitoring is feasible in terrain where field access is difficult and traditional data telemetry services (i.e. the cellular network) are not available. In particular, the low cost of production and portability of monitoring components make the system suitable for rugged, remote terrain. The very low power consumption also allows for extended deployment with only a handful of small lithium batteries. This creates opportunity and technology to monitor hillslope hydrology in remote communities that previously could not have accesses life-saving monitoring equipment due to the prohibitive costs of the instruments and installation. It is important to note, however, that LoRa transmission strength in the Sitka application did not meet theoretical calculations. In the Sitka network, variable topography, air humidity, and forest density likely limited the range of reliable LoRa transmission (Fig. 24). Sites within 2–2.5 km of the receiving hub with a clear line of sight successfully transmitted data packets. In locations where signal strength was unreliable, a directional antenna or increased antenna height might improve signal strength.

An example of the relationships between rainfall and hillslope hydrology is shown in Fig. 23. For example, following rain events on August 28, 2020, volumetric water content and water pressure both increased. After rainfall ended, the soils returned to background moisture levels over the course of hours to days. Notably, not all sensors performed reliably at all monitoring sites, potentially due to faulty sensors, limited range of measurement, or other connection issues (Fig. 24). For example, the STEMMA sensors appear to be outside of the range of measurement at five of the six node sites. This could be caused by the extremely dense, fine-grained soils of the study area (clay-rich till and volcanic soil) and perennial saturation at many of the monitoring sites [25]. The Teros sensors performed more reliably, with 12/18 sensors providing reliable water content data that responded to rainfall. These limitations demonstrate the importance of redundancy of monitoring equipment within sites and at multiple sites, including the two brands of soil moisture sensors which performed best under slightly different conditions.

Additionally, the accelerometer, which was intended to trigger an alarm status when a threshold was passed, initiated multiple false alarms at several sites. In the alarm state, nodes transmitted data as quickly as possible (multiple times per second). To prevent this error and preserve battery life, the accelerometers were disabled after the initial deployment. Although theoretical battery life is >6 months (Appendix A), maximum observed battery life was 2–3 months in the Sitka application, likely due to cold temperatures (−6 to 6 °C at the high-elevation sites in November-January 2020–2021). A less frequent observation/transmission rate (10–15 min) would extend battery life for remote deployments.

Long term observations at these locations will improve the understanding of landslide initiation in the spatially heterogeneous landscape of Sitka. Machine learning of the complex data stream from this system provides a potential means to advance landslide prediction and risk mitigation [26], [27]. As shown in Fig. 23, both soil moisture and groundwater pressure respond to rainfall. These relationships can be used to determine threshold rainfall and soil characteristics that promote landsliding. Community planners and individuals can use the real-time observations from this system to take life-saving action before landslides occur.

## Human and animal rights

The work does not use any human or animal subjects.

## Funding

This work is supported by the USDA National Institute of Food and Agriculture, Hatch project NI18HFPXXXXXG055, the National Science Foundation award #1832170 and #1831770.

## Declaration of Competing Interest

The authors declare that they have no known competing financial interests or personal relationships that could have appeared to influence the work reported in this paper.

## Figures and Tables

**Table A1:** Current draw of node during operating phases.

Operation Mode	Measured Current Draw (mA)	Time Period (seconds)
Wake up and measure sensors	40	2
Lora transmit	150	0.75
SD card recording	7	2
Deep sleep	<1	300

## Appendix A Power consumption calculations

Battery self-discharge: ~5% of battery capacity per month

Battery life calculated using two batteries with 6600mAh capacity each.

$$\mathrm{BatterySelfDischargeRate}=\frac{\mathrm{SelfDischargeRatePerMonth}}{730}$$

$$\mathrm{BatterySelfDischargeRate}=\frac{0.05*13200}{730}=0.904mA$$

$$\mathrm{CurrentDraw}=\frac{\sum CurrentDraw*TimePeriod}{\sum TimePeriod}+SelfDischargeRate$$

$$\mathrm{CurrentDraw}=\frac{\left(40*2\right)+\left(150*0.75\right)+\left(7*2\right)+\left(1*300\right)}{2+0.75+2+300}+0.904=2.566mA$$

$$\mathrm{BatteryLife}=\frac{\mathrm{BatteryCapacity}}{\mathrm{CurrentDraw}}$$

$$\mathrm{BatteryLife}=\frac{2*6600}{2.566}=5144hours=214.3days=7.03months$$
