# Supplementary material for: SitkaNet: A low-cost, distributed sensor network for landslide monitoring and study
Source: HardwareX. 2021 Mar 11;9:e00191. doi: 10.1016/j.ohx.2021.e00191 (PMC9041236; doi:10.1016/j.ohx.2021.e00191)
Supplement: Supplementary data 1 [file mmc1.docx]

All design files have been uploaded to OSF:

[*https://doi.org/10.17605/OSF.IO/497GT*](https://doi.org/10.17605/OSF.IO/497GT)
